# Digital financial literacy and trust in hybrid AI advisory: Explaining perceived private wealth management capability among digitally engaged retail investors in an emerging market

**DOI:** 10.1371/journal.pone.0353922

**Published:** 2026-10-30

**Authors:** Minh Tu Tran, Marie Paseková

**Affiliations:** 1 Ho Chi Minh City University of Economics and Finance, Ho Chi Minh City, Vietnam; 2 Tomas Bata University in Zlín, Zlín, Czech Republic; Universiti Pendidikan Sultan Idris, MALAYSIA

## Abstract

This study investigates how digital financial literacy, perceived transparency of artificial intelligence advisory services, investment and wealth knowledge, and cyber-risk self-protection shape Perceived Private Wealth Management Capability (PWMC) among digitally engaged retail investors in an emerging-market context. PWMC is conceptualized as a self-assessed capability outcome rather than an objective indicator of wealth-management performance, portfolio returns, or private-banking status. Drawing on the financial capability framework, trust in automation theory, and protection motivation theory, the study develops a trust-to-capability model in which trust in hybrid artificial intelligence advisory and digital wealth management behavior serves as a key explanatory mechanism. A non-probability sample of 249 digitally engaged retail investors in Vietnam was analyzed using partial least squares structural equation modeling in SmartPLS 4.1, with 5,000 bootstrap subsamples. The results show that digital financial literacy is the strongest antecedent of trust in hybrid artificial intelligence advisory (β = 0.517, p < 0.001). At the same time, perceived advisory transparency also exerts a significant positive effect (β = 0.261, p < 0.001). Investment and wealth knowledge emerged as the strongest predictor of digital wealth management behavior (β = 0.348, p < 0.001). Trust, digital wealth management behavior, and investment and wealth knowledge each positively influence perceived private wealth management capability. Digital wealth management behavior partially mediates the effects of trust, investment, and wealth knowledge on perceived capability. Cyber-risk self-protection limited evidence of a boundary condition by strengthening the relationship between trust and digital wealth management behavior. However, the effect size is small and should be interpreted cautiously (β = 0.098, p = 0.038). The model explains 60.2% of the variance in digital wealth management behavior and 44.3% of the variance in perceived private wealth management capability. By moving beyond conventional explanations of robo-advisory adoption and technology acceptance, this study theorizes hybrid AI advisory as a calibrated trust environment in which retail investors’ perceived capability depends not only on whether they trust AI-supported advice but also on whether that trust translates into protective, enacted digital wealth management behavior. The findings should be interpreted in the context of digitally engaged retail investors. They should not be generalized to private banking clients, high-net-worth investors, objective portfolio performance, or verified wealth outcomes.

## 1 Introduction

The digital transformation of financial services has accelerated substantially in recent years, reshaping how individuals make investment decisions and manage their personal wealth. Advanced digital tools, particularly those powered by artificial intelligence (AI), are now integral components of modern wealth management systems, enabling personalized portfolio guidance, automated rebalancing, and scalable advisory services that were previously accessible only to institutional investors. However, this technological revolution also introduces new cognitive and behavioral challenges: while digital platforms promise efficiency and broader access, they simultaneously require users to possess a blend of financial knowledge, technological competence, and trust in algorithmic outputs to fully realize these benefits.

Recent empirical studies indicate that digital financial literacy, the ability to understand and apply financial knowledge in digitally mediated environments, has become a foundational competence for effective engagement with digital financial services [1]. Moreover, trust dynamics within hybrid AI advisory systems remain pivotal determinants of user engagement and decision quality; behavioral research shows that investor trust significantly shapes how retail clients interact with AI advisors and incorporate algorithmic recommendations into real-world investment decisions [2]. Emerging evidence also emphasizes the role of transparency and interpretability as mechanisms to reduce uncertainty and enhance trust in robo-advisory contexts, where recommendations may be complex and difficult for users to evaluate [3,4]. Although recent studies consistently highlight the importance of digital financial literacy and transparency in strengthening trust in AI-supported financial services, empirical evidence remains fragmented on how such trust translates into actual wealth-management behaviors. Existing research has largely focused on technology acceptance and intention to use, while the behavioral pathway through which trust contributes to investors’ capability development remains insufficiently understood.

Although prior research has advanced understanding of digital financial literacy, robo-advisory adoption, AI transparency, and trust in automated systems, three important limitations remain. First, much of the robo-advisory literature conceptualizes investors primarily as adopters of automated advisory platforms, with attention given to perceived usefulness, trust, risk, and intention to use. This perspective is valuable, but it does not fully capture the hybrid advisory context in which investors must evaluate recommendations generated through the combined roles of artificial intelligence and human expertise. Hybrid AI advisory, therefore, poses a different theoretical problem from conventional technology adoption: the key issue is not only whether users accept the advisory system, but whether they develop calibrated trust and translate such trust into responsible wealth management actions.

Second, trust in automation research emphasizes that trust is most consequential under uncertainty and complexity, where users cannot fully verify system processes and must decide whether reliance is appropriate. In AI-supported wealth management, however, trust is theoretically ambivalent. Calibrated trust may help investors interpret advisory recommendations and use them more effectively, whereas excessive or poorly calibrated trust may encourage passive compliance, over-reliance, or insufficient monitoring. Existing research has paid considerable attention to how transparency and explainability shape trust in AI systems. Still, little is known about how trust becomes enacted in digital portfolio monitoring, rebalancing, and advisory-based decision-making. This leaves the trust-action pathway in AI-enabled wealth management underdeveloped.

Third, financial capability research has long emphasized that financial knowledge becomes meaningful when it is translated into behavior. Yet, in digital wealth management, the conversion of knowledge and trust into action may also depend on users’ perceived ability to protect themselves against cyber risks. Investors may trust a hybrid AI advisory system yet hesitate to act if they feel unable to recognize threats, secure their accounts, or manage digital financial risks. Cyber-risk self-protection should therefore be theorized as a potential boundary condition that helps explain when trust becomes actionable, rather than as the central mechanism of the trust-to-capability process.

This study addresses these gaps by developing and testing a trust-to-capability model of hybrid AI advisory in an emerging-market context. The model links digital financial literacy, perceived AI advisory transparency, trust in hybrid AI advisory, investment and wealth knowledge, cyber-risk self-protection, and digital wealth management behavior to explain Perceived Private Wealth Management Capability (PWMC) among digitally engaged retail investors. PWMC is conceptualized as a perceived, self-assessed capability outcome rather than as objective wealth performance or evidence of private-banking capability. The study makes three contributions. First, it extends the financial capability framework by conceptualizing perceived PWMC as a capability outcome shaped by both knowledge-based resources and enacted digital wealth management behavior, rather than as a direct consequence of financial knowledge alone. Second, it extends trust in automation theory by shifting the focus from trust formation and technology acceptance to the conversion of calibrated trust into digital wealth management behavior. Third, it extends protection motivation theory by positioning cyber-risk self-protection as a modest protective boundary condition that supports, but does not dominate, the trust-behavior relationship.

In this study, hybrid AI advisory is operationally defined as a wealth-management service configuration in which artificial intelligence generates or supports investment recommendations. At the same time, human advisors provide complementary interpretation, oversight, or personalized guidance. This definition distinguishes hybrid AI advisory from fully automated robo-advisory systems and ensures that respondents evaluated a technology-enabled advisory context involving both algorithmic and human elements.

Accordingly, the primary objective of this study is to explain how user-side capability, system-side transparency, investment and wealth knowledge, and cyber-risk self-protection jointly shape perceived private wealth management capability through trust and enacted digital wealth management behavior. Specifically, the study examines (i) the antecedents of trust in hybrid AI advisory, (ii) the direct and mediated pathways linking trust and investment and wealth knowledge to perceived capability through digital wealth management behavior, and (iii) whether cyber-risk self-protection conditions the trust-to-behavior relationship. The Financial Capability Framework, Trust in Automation Theory, and Protection Motivation Theory are used respectively to explain the knowledge-behavior-capability pathway, informed reliance on hybrid AI advisory, and the proposed protective boundary condition.

## 2 Literature review

### 2.1 Theoretical background

This study is grounded in three complementary perspectives: the financial capability framework, trust in automation theory, and protection motivation theory. Their integration is necessary because private wealth management in a digital environment cannot be explained solely by financial knowledge or access to technology. Individuals must be able to understand financial information, evaluate technology-enabled recommendations, translate knowledge into consistent wealth management behavior, and protect themselves against cyber risks. The proposed framework, therefore, conceptualizes PWMC as a perceived capability outcome shaped by the interaction of knowledge-based, technological, behavioral, and protective mechanisms.

#### 2.1.1 Financial capability framework in the digital wealth management context.

The financial capability framework provides the primary foundation for explaining how individuals develop the ability to manage personal wealth effectively. Financial capability extends beyond the possession of financial knowledge. It reflects the capacity to apply relevant knowledge, skills, attitudes, and behaviors to make financial decisions and manage resources over time [5]. Similarly, financial literacy is defined as a combination of awareness, knowledge, skills, attitudes, and behaviors required to make sound financial decisions and achieve individual financial well-being.

The distinction between knowledge and enacted behavior is particularly important in wealth management [6]. argues that financial capability develops through an interconnected process in which financial knowledge contributes to behavioral change and, subsequently, to improved financial outcomes. Knowledge alone may not lead to effective financial management unless it is translated into systematic actions. In the present study, investment and wealth knowledge represent an individual’s understanding of financial products, investment strategies, diversification, and risk–return trade-offs. Digital wealth management behavior captures the application of such knowledge through activities such as monitoring portfolios, adjusting asset allocations, using digital financial tools, and acting on advisory recommendations.

The digitalization of financial services adds a further layer to the financial capability framework. Digital financial literacy is not merely conventional financial literacy delivered through online channels. It also involves the ability to assess digital financial services, understand their opportunities and risks, and use digital technologies safely and effectively [7,8]. This distinction is especially relevant to private wealth management, where investors increasingly interact with digital platforms and technology-supported advisory systems. Recent evidence also suggests that digital financial literacy influences financial behavior by enabling individuals to use digital tools more deliberately in managing savings and spending decisions [9].

Accordingly, the financial capability framework supports the proposed relationships between investment and wealth knowledge, digital wealth management behavior, and perceived private wealth management capability. It also provides a theoretical basis for examining digital wealth management behavior as a mediating mechanism through which knowledge is converted into a broader perceived capability to manage personal wealth.

#### 2.1.2 Trust in automation theory and hybrid artificial intelligence advisory.

Trust in automation theory explains why individuals rely on technological systems when making complex decisions [10]. define trust in automation as an attitude that an automated system will help an individual achieve their goals in situations characterized by uncertainty and vulnerability. Trust becomes especially important when users cannot independently verify every process or recommendation generated by a complex system. In such circumstances, trust influences whether users appropriately rely on automated support rather than reject, ignore, or overuse it.

This perspective is relevant to hybrid artificial intelligence advisory, in which recommendations are generated through the combined contributions of artificial intelligence and human expertise. The hybrid structure may improve advisory efficiency and personalization, but it also creates a reliance problem. Investors must decide whether the system is sufficiently reliable, competent, and understandable before incorporating its recommendations into wealth management decisions. Trust, therefore, serves as a central psychological mechanism linking users’ evaluation of the advisory system to their subsequent financial actions.

The literature on trust in artificial intelligence further indicates that trust is shaped by both user-related and system-related factors [11]. Digital financial literacy represents a user-related antecedent because financially literate users are better positioned to assess the relevance, credibility, and limitations of technology-enabled recommendations. Greater literacy should not be interpreted as unconditional acceptance of artificial intelligence. Rather, it enables informed, calibrated trust when users perceive the advisory system as credible and suitable for their financial objectives.

Perceived transparency of artificial intelligence advisory systems represents a system-related antecedent of trust. In the present context, transparency refers to the extent to which users perceive that advisory recommendations are understandable, sufficiently explained, and open to evaluation [12]. demonstrates that explainability and causability shape trust and subsequent user responses by allowing individuals to assess how artificial intelligence systems generate recommendations. Transparency should therefore be understood as a mechanism to support trust calibration rather than to encourage blind reliance. When users can interpret the rationale underlying advisory outputs, uncertainty is reduced, and informed reliance becomes more likely.

Trust may influence perceived private wealth management capability through two complementary channels. First, it can directly support investors’ willingness to incorporate credible advisory insights into their decision-making processes. Second, it can indirectly improve perceived capability by encouraging more consistent digital wealth management behavior. This logic explains why digital wealth management behavior is positioned as a mediator between trust in hybrid artificial intelligence advisory and perceived private wealth management capability.

#### 2.1.3 Protection motivation theory and cyber-risk self-protection.

Protection motivation theory provides the theoretical foundation for examining cyber-risk self-protection as a boundary condition in digital wealth management. The theory was originally developed to explain how individuals respond to perceived threats by assessing both the seriousness of a threat and their ability to implement an effective protective response [13]. Its later development emphasized the role of coping appraisal, including response efficacy and self-efficacy, in determining whether individuals adopt preventive behaviors [14].

Protection motivation theory has become an established framework for analyzing information security behavior. Research in information systems demonstrates that users’ security-related actions depend not only on their awareness of threats but also on their perceived capacity to cope with those threats effectively [15,16]. In digital wealth management, these considerations are particularly important because financial platforms may expose users to phishing, account compromise, data misuse, and other cybersecurity threats.

Cyber-risk self-protection refers to an individual’s ability to recognize, prevent, and mitigate digital security risks when using financial platforms. This construct is not treated merely as another independent predictor of digital behavior. Instead, it is conceptualized as a potential boundary condition that may influence the extent to which trust translates into action. Even when investors trust a hybrid artificial intelligence advisory system, they may hesitate to implement its recommendations if they feel unable to protect their accounts or manage cyber threats. Conversely, investors with stronger cyber-risk self-protection may be more willing to act on trusted recommendations because they feel capable of engaging safely with digital platforms.

Accordingly, protection motivation theory supports examining cyber-risk self-protection as a potential moderating condition in the relationship between trust in hybrid artificial intelligence advisory and digital wealth management behavior.

#### 2.1.4 Integrated theoretical framework.

The integration of the three perspectives provides a coherent explanation of perceived private wealth management capability in a digitally mediated environment. The financial capability framework explains how investment and wealth knowledge are translated into perceived capability, both directly and through digital wealth management behavior. Trust in automation theory explains why digital financial literacy and perceived transparency of artificial intelligence advisory shape trust in hybrid artificial intelligence advisory, and why that trust subsequently affects behavior and perceived capability. Protection motivation theory introduces a cybersecurity-related boundary condition by explaining why the behavioral influence of trust may vary depending on investors’ perceived cyber-risk self-protection.

The proposed framework, therefore, distinguishes between four theoretically meaningful components: knowledge-based resources, perceived technological trustworthiness, enacted digital behavior, and protective capability. This integration offers a parsimonious foundation for examining how individuals develop perceived private wealth management capability when using hybrid artificial-intelligence advisory services in an emerging-market context.

### 2.2 Related Empirical studies

#### 2.2.1 Digital capability and informed trust.

Digital financial literacy has increasingly been recognized as a necessary capability for individuals who use digital financial services. Unlike conventional financial literacy, digital financial literacy combines financial knowledge with the ability to access, evaluate, and use digital financial tools while recognizing the risks associated with digitally mediated transactions [7]. This distinction is important in digital wealth management because investors are not only required to understand financial products and investment principles, but also to interpret information delivered through mobile platforms, digital investment applications, robo-advisory interfaces, and AI-supported advisory systems.

Recent empirical evidence suggests that digital financial literacy is associated with stronger financial well-being and safer engagement with digital financial services [17]., for example, show that digital financial literacy is related to financial well-being and that this relationship is partly explained by users’ financial knowledge and ability to protect themselves against digital fraud. This evidence suggests that digital financial literacy should not be understood merely as technical familiarity with digital platforms. Rather, it represents a broader user capability that enables individuals to evaluate digital financial information, recognize risks, and make more informed decisions.

In the context of AI-supported wealth management, digital financial literacy may also shape trust formation. Trust in AI is shaped not only by system design but also by user-related factors, including users’ ability to understand and evaluate the system’s outputs [11]. Digitally literate investors may be better positioned to assess whether AI-supported recommendations are credible, relevant, and consistent with their financial objectives. Therefore, digital financial literacy contributes to informed trust rather than blind acceptance. This is particularly relevant in hybrid AI advisory, where investors must interpret recommendations generated through both algorithmic analysis and human advisory input.

However, existing studies on digital financial literacy have primarily focused on financial inclusion, use of digital financial services, financial well-being, or general digital financial behavior. They provide important evidence that digital financial literacy matters, but they offer a limited explanation of how digital financial literacy contributes to trust in hybrid AI advisory and how such trust is subsequently converted into perceived private wealth management capability. This study addresses this limitation by positioning digital financial literacy as a user-related antecedent of trust in hybrid AI advisory within a broader trust-to-capability model.

#### 2.2.2 AI transparency and calibrated reliance.

AI transparency and explainability have become central issues in research on artificial intelligence adoption and human-AI interaction. Prior studies indicate that users are more likely to trust and accept AI systems when they can understand the logic, rationale, or explanation underlying algorithmic outputs [12]. Similarly, research on AI transparency suggests that explanations can improve perceived legitimacy by helping users understand why an AI-based decision or recommendation has been produced [18]. In financial advisory contexts, this issue is especially important because investment recommendations often involve uncertainty, risk preferences, and long-term financial consequences.

The robo-advisory literature has shown that automated advisory services can increase access to financial advice, reduce service costs, and support portfolio allocation through algorithmic decision rules [19]. Other studies have argued that robo-advisors may help mitigate certain behavioral biases among retail investors by providing structured, data-driven recommendations [20]. More recent work on AI-enabled robo-advisory services also suggests that investors may perceive robo-advisors as useful, but may still expect human involvement when decisions involve judgment, emotions, or complex personal circumstances [21]. These findings are directly relevant to hybrid AI advisory, where AI-supported recommendations are combined with human expertise.

Recent evidence on retail investors’ interactions with AI financial advisors likewise indicates that trust dynamics are associated with behavioral responses and decision quality [2].

Nevertheless, much of the robo-advisory literature remains adoption-oriented. It explains whether investors are willing to use automated advisory platforms, but it provides less insight into whether AI-supported advice leads to responsible digital wealth management behavior. In addition, transparency is often treated as a predictor of trust or acceptance, rather than as part of a broader calibrated reliance process. Trust in automation theory emphasizes that trust should support appropriate, not excessive or insufficient, reliance [10]. In other words, the key theoretical issue is not simply whether transparency increases trust, but whether transparency enables users to rely on AI-supported recommendations in an informed and controlled manner.

This distinction matters in private wealth management. Investors may trust a recommendation because it appears transparent, but transparency alone does not guarantee that they will monitor portfolios, rebalance assets, evaluate risk-return implications, or implement recommendations responsibly. The current study, therefore, extends prior work by examining perceived AI advisory transparency as a system-related antecedent of trust, and by testing whether trust translates into digital wealth management behavior and perceived private wealth management capability.

#### 2.2.3 Behavioral enactment in financial capability.

The financial capability literature emphasizes that knowledge alone is insufficient for effective financial outcomes. Financial capability involves the ability to apply knowledge, skills, attitudes, and behavior in financial decision-making [6,22]. This knowledge-behavior distinction is important because individuals may possess financial knowledge but fail to convert it into consistent financial practices. In wealth management, such practices include monitoring investment portfolios, adjusting asset allocations, using digital advisory tools, comparing investment options, and making disciplined decisions over time.

Prior studies on financial literacy and investment behavior generally support the view that financially knowledgeable individuals are more likely to participate in financial markets, evaluate investment opportunities, and make more informed financial decisions [23,24]. However, in digital wealth management, investment and wealth knowledge must be translated into platform-mediated behavior. Investors need to use digital tools, interpret portfolio information, respond to advisory recommendations, and act consistently under uncertainty. Therefore, digital wealth management behavior represents an important mechanism by which knowledge becomes capability.

This behavioral mechanism is also relevant to trust in hybrid AI advisory. Trust may encourage investors to consider AI-supported recommendations, but trust alone does not automatically produce stronger wealth management capability. The value of trust depends on whether it prompts investors to take concrete financial actions, such as monitoring portfolios, reviewing advisory suggestions, and making informed adjustments. Therefore, digital wealth management behavior should be theorized as a mediator rather than merely as another outcome variable.

Existing studies on robo-advisory and fintech adoption often focus on intention to use, platform acceptance, perceived usefulness, or general engagement. While these outcomes are useful, they do not fully capture the behavioral process through which investors develop perceived private wealth management capability. This study addresses this gap by positioning digital wealth management behavior as the mechanism through which both trust in hybrid AI advisory and investment and wealth knowledge are converted into perceived private wealth management capability.

#### 2.2.4 Cyber-risk self-protection as a boundary condition.

Digital wealth management also exposes investors to cybersecurity risks, including phishing, account compromise, unauthorized access, identity misuse, and fraudulent investment communications. In this context, users’ ability to protect themselves becomes an important condition for safe and confident participation in digital financial services. Protection Motivation Theory explains protective behavior through threat appraisal and coping appraisal, particularly individuals’ perceptions of response efficacy and self-efficacy [13,14]. In information security research, coping appraisal has been shown to play an important role in motivating security-related behavior [16].

Cyber-risk self-protection is therefore relevant not only as a security behavior but also as a capability that may influence whether trusted digital advice becomes actionable. Investors may trust a hybrid AI advisory system but still hesitate to implement recommendations via digital platforms if they feel unable to recognize threats, protect their accounts, or respond to suspicious activity. Conversely, investors with stronger cyber-risk self-protection may feel more capable of acting on trusted advisory recommendations because they perceive digital engagement as manageable and controllable.

Prior information security studies have mainly examined cyber-risk awareness, threat perception, self-efficacy, or security behavior as direct predictors of protective actions. These studies provide important insights into why users adopt security practices, but they rarely examine cybersecurity capability as a moderator of the relationship between trust and financial behavior. This is a meaningful limitation in AI-enabled wealth management because trust and cyber-risk exposure coexist. A user may trust the advisory recommendation but still avoid action if the digital environment feels unsafe.

Accordingly, this study conceptualizes cyber-risk self-protection as a boundary condition in the trust-behavior relationship. This approach extends Protection Motivation Theory into the digital wealth management context by arguing that protective capability may condition, rather than dominate, the conversion of trust in hybrid AI advisory into digital wealth management behavior.

#### 2.2.5 Synthesis of prior literature and positioning of the present study.

The reviewed literature provides important foundations for understanding digital financial literacy, AI transparency, trust in automation, financial capability, robo-advisory adoption, and cybersecurity behavior. However, these streams of research remain partly fragmented. Digital financial literacy studies show that users need both financial and digital competence, but they rarely examine how such competence shapes trust in hybrid AI advisory. Robo-advisory studies explain adoption and platform use, but they often stop short of examining whether trust is converted into responsible digital wealth management behavior. Financial capability research emphasizes the importance of behavior, but has paid less attention to AI-supported advisory environments. Information security research explains protective motivation, but has not sufficiently considered cyber-risk self-protection as a boundary condition in the trust-action process.

The present study integrates these streams by developing a trust-to-capability model of hybrid AI advisory. The model proposes that digital financial literacy and perceived AI advisory transparency shape trust in hybrid AI advisory; trust and investment and wealth knowledge promote digital wealth management behavior; digital wealth management behavior converts trust and knowledge into perceived private wealth management capability; and cyber-risk self-protection provides a modest boundary condition in the relationship between trust and behavior. In doing so, the study moves beyond a simple adoption-based explanation and examines how digitally engaged retail investors transform AI-supported trust into responsible digital wealth management action.

### 2.3 Research gap

Despite growing scholarly attention to digital financial literacy, robo-advisory services, artificial intelligence transparency, and trust in automated systems, the literature still offers an incomplete explanation of how trust in hybrid AI advisory translates into perceived private wealth management capability. Prior research on robo-advisory has largely emphasized the digitalization and automation of financial advisory services, platform adoption, and the potential of robo-advisors to reduce entry barriers and mitigate behavioral biases among retail investors [19–21]. While these studies provide important insights into automated advisory services, they tend to conceptualize investors primarily as adopters or users of digital advisory platforms. They pay less attention to the behavioral process by which trust in AI-supported advice translates into ongoing wealth management actions, such as portfolio monitoring, advisory-based adjustments, and disciplined digital financial decision-making.

A second limitation concerns the treatment of trust in AI and automation research. Trust in automation theory emphasizes that trust is especially important when users face uncertainty and cannot fully understand or verify automated processes [10]. Similarly, research on human trust in artificial intelligence shows that trust is shaped by both user-related and system-related factors, including users’ competence, the form of AI representation, and perceived system capability [11]. However, trust in automation should not be interpreted as inherently beneficial. The central theoretical issue is appropriate or calibrated reliance, because under-trust may lead users to ignore useful advice, whereas over-trust may produce passive compliance and insufficient monitoring [10]. In AI-supported wealth management, this distinction is critical because investors must not only trust advisory recommendations but also evaluate, monitor, and implement them responsibly.

A third limitation relates to transparency and explainability. Prior studies suggest that AI transparency, explainability, and causability can improve users’ trust and acceptance by making algorithmic outputs more understandable and interpretable [12,18]. However, explainability alone does not ensure responsible financial action. Users may understand an AI-generated recommendation but still fail to apply it effectively if they lack digital financial literacy, investment knowledge, or confidence in using digital platforms safely. Digital financial literacy has therefore become increasingly important because it includes not only financial knowledge, but also the ability to access, evaluate, and use digital financial services while understanding their risks [7,17].

A fourth limitation concerns the role of cybersecurity capability in AI-enabled financial behavior. Protection motivation theory has been widely used to explain why individuals adopt information security behaviors, particularly through threat and coping appraisals [13,14]. Recent meta-analytic evidence in information security research further shows that response efficacy and self-efficacy are important predictors of security-related motivation and behavior [16]. However, in digital wealth management, cyber-risk self-protection may play a broader theoretical role. It may not only predict security behavior, but also determine whether investors feel sufficiently capable of acting on trusted AI-supported financial advice. A user may trust a hybrid AI advisory system but still hesitate to act if they feel unable to recognize digital threats, secure their accounts, or manage cyber risk exposure.

Accordingly, this study addresses these gaps by developing a trust-to-capability model of hybrid AI advisory in an emerging-market context. The model integrates digital financial literacy and perceived AI advisory transparency as antecedents of trust, digital wealth management behavior as the mechanism through which trust and investment knowledge are converted into perceived private wealth management capability, and cyber-risk self-protection as a modest boundary condition that may support the trust-behavior relationship. By doing so, the study moves beyond conventional research on robo-advisory adoption. It explains not only whether digitally engaged retail investors trust AI-supported advisory systems, but also how and under what protective conditions that trust translates into responsible digital wealth management behavior.

The central unresolved issue is therefore not simply whether retail investors adopt or trust AI-supported advisory services, but how that trust translates into capability-relevant behavior and under what protective conditions this conversion is more likely to occur. Addressing this process gap connects previously separate research on digital financial literacy, AI trust and transparency, financial capability, and cybersecurity into a single testable framework.

### 2.4 Research hypotheses

Digital financial literacy refers to an individual’s ability to access, understand, and apply financial knowledge in digital contexts, including online investment platforms, mobile banking applications, and other digital financial services. Individuals with higher digital financial literacy can critically evaluate complex financial products and digital tools, enhancing their ability to trust hybrid advisory systems that integrate artificial intelligence and human expertise. Prior studies indicate that digital financial literacy significantly influences trust in digital financial services because literate users can assess the reliability and transparency of online advisory tools [7,8,17,23]. Therefore, digital financial literacy is expected to impact trust in hybrid artificial-intelligence advisory services positively.

***H1:*** *Digital financial literacy positively affects trust in hybrid artificial-intelligence advisory services.*

Perceived artificial intelligence advisory transparency is defined as the degree to which users perceive the AI-driven decision-making process and recommendations as understandable, open, and explainable. Transparency allows users to comprehend how AI suggestions are generated and to assess their reliability, which is crucial for fostering trust. Research in algorithmic decision-making suggests that perceived transparency significantly strengthens trust in AI-enabled services by reducing perceived risk and uncertainty [3,12,18]. Therefore, individuals who perceive greater transparency in AI advisory systems are likely to exhibit greater trust in hybrid AI advisory services.

***H2:*** *Perceived transparency of artificial intelligence advisory positively affects trust in a hybrid artificial intelligence advisory.*

Trust in hybrid artificial intelligence advisory refers to the belief that advisory services combining AI and human expertise are reliable, competent, and consistent in delivering accurate recommendations. Digital wealth management behavior represents the actions individuals take to monitor, manage, and adjust their financial portfolios using digital tools, such as rebalancing investments, tracking asset performance, and executing transactions online. Studies show that trust in digital advisory services positively influences user engagement and behavioral compliance, as users who trust the platform are more likely to follow recommendations and manage their wealth proactively [25,26]. Consequently, trust in hybrid AI advisory is hypothesized to enhance digital wealth management behavior.

***H3:*** *Trust in hybrid artificial-intelligence advisory positively affects digital wealth-management behavior.*

Investment and wealth knowledge refers to an individual’s understanding of financial products, investment strategies, portfolio diversification, risk-return trade-offs, and long-term wealth accumulation. Individuals with higher levels of investment and wealth knowledge are better able to evaluate financial opportunities and risks, which enables them to make informed decisions and engage more actively in digital wealth management activities. Prior research has shown that financial knowledge positively influences financial behavior, including portfolio monitoring and investment decision-making in digital contexts [24,27]. Thus, the fourth hypothesis is proposed:

***H4:*** *Investment and wealth knowledge positively affect digital wealth management behavior.*

Trust in hybrid artificial intelligence advisory may directly enhance perceived private wealth management capability because trust reduces uncertainty and enables investors to incorporate AI-supported advisory input into their financial judgment. In hybrid advisory settings, users do not rely solely on algorithmic output or human expertise; rather, they evaluate recommendations generated through the combined contribution of artificial intelligence and human advisory support. Trust in automation theory suggests that trust is particularly important when users face complex systems whose processes cannot be fully observed or independently verified [10]. However, trust should not be understood as unconditional acceptance. The key theoretical concern is calibrated reliance, whereby users rely on advisory support to the extent that it is perceived as competent, reliable, and appropriate for the decision context [10,11]. In private wealth management, calibrated trust may strengthen investors’ perceived capability by helping them interpret advisory recommendations, compare options, evaluate portfolio implications, and make more confident financial decisions.

At the same time, trust in hybrid AI advisory can pose risks if it becomes excessive or poorly calibrated. When investors over-trust AI-supported advice, they may passively accept recommendations, reduce independent evaluation, or neglect portfolio monitoring. This concern is especially relevant in wealth management because investment decisions involve uncertainty, changing market conditions, and individual risk preferences. Therefore, the expected positive effect of trust on perceived private wealth management capability is theoretically justified only when trust supports informed reliance rather than blind compliance. In the present study, trust in hybrid AI advisory is conceptualized as a form of informed confidence in the advisory system’s reliability and competence. Accordingly, investors who trust hybrid AI advisory are expected to perceive themselves as more capable of managing personal wealth effectively.

***H5*** *: Trust in hybrid artificial intelligence advisory positively affects perceived private wealth management capability.*

Digital wealth management behavior represents the behavioral enactment of financial capability in a digitally mediated environment. Financial capability research emphasizes that knowledge and attitudes become meaningful when individuals translate them into financial behaviors such as planning, monitoring, controlling, and adjusting financial decisions [6,27]. In the context of digital wealth management, such behavior includes monitoring digital investment portfolios, using online financial tools, evaluating advisory recommendations, and adjusting asset allocation when necessary. These actions allow investors to move beyond passively receiving information and actively manage their personal wealth.

Digital wealth management behavior is therefore expected to strengthen perceived private wealth management capability because repeated engagement with digital financial tools helps investors develop greater control, discipline, and confidence in managing financial resources. Investors who actively monitor portfolios and use advisory information are more likely to understand their financial position, detect changes in risk exposure, and respond to investment opportunities or threats. This behavioral pathway is particularly important in AI-supported advisory environments, where the value of advisory recommendations depends on whether users transform advice into concrete financial action. Accordingly, digital wealth management behavior is expected to affect perceived private wealth management capability positively.

***H6*** *: Digital wealth management behavior positively affects perceived private wealth management capability.*

Investment and wealth knowledge may also directly affect perceived private wealth management capability, even when digital wealth management behavior is considered a mediating mechanism. Financial knowledge provides investors with the cognitive foundation needed to understand financial products, evaluate risk-return trade-offs, interpret portfolio information, and assess the suitability of advisory recommendations. Prior research shows that financial literacy is associated with stock market participation and more informed financial decision-making [23,24]. In private wealth management, investment and wealth knowledge enable individuals to make sense of financial information and assess whether a recommended strategy aligns with their goals, risk tolerance, and long-term wealth accumulation plans.

The direct effect of investment and wealth knowledge is theoretically important because not all aspects of capability are fully mediated through observable digital behavior. Knowledge may improve perceived capability by strengthening strategic judgment, financial confidence, risk awareness, and the ability to evaluate complex investment choices before taking action. In hybrid AI advisory settings, knowledgeable investors are also better positioned to question, interpret, or contextualize AI-supported recommendations rather than accepting them mechanically. Therefore, investment and wealth knowledge are expected to contribute directly to perceived private wealth management capability and indirectly support capability through digital wealth management behavior.

***H7*** *: Investment and wealth knowledge positively affect perceived private wealth management capability.*

Digital wealth management behavior is expected to mediate the relationship between trust in hybrid artificial intelligence advisory and perceived private wealth management capability. Trust may increase investors’ willingness to consider AI-supported recommendations, but it alone does not necessarily yield stronger perceived capability unless it translates into active wealth management practices. Trust in automation theory suggests that appropriate reliance becomes meaningful when users incorporate technological support into task performance [10]. In the present context, this means that investors must convert trust into behaviors such as portfolio monitoring, advisory-based adjustment, and disciplined use of digital financial tools. Therefore, trust in hybrid AI advisory is expected to enhance perceived private wealth management capability, in part through digital wealth management behavior.

***H8:*** *Digital wealth management behavior mediates the relationship between trust in hybrid artificial intelligence advisory and perceived private wealth management capability.*

Digital wealth management behavior is also expected to mediate the relationship between investment and wealth knowledge and perceived private wealth management capability. The financial capability literature emphasizes that knowledge is necessary but insufficient unless it is enacted through financial behavior [6]. Investors with stronger investment and wealth knowledge may better understand financial products and risk-return trade-offs. Still, such knowledge contributes more strongly to perceived capability when applied through portfolio monitoring, use of digital tools, and informed adjustments to financial decisions. Accordingly, digital wealth management behavior represents the behavioral mechanism through which investment and wealth knowledge is converted into perceived private wealth management capability.

***H9:*** *Digital wealth management behavior mediates the relationship between investment, wealth knowledge, and perceived private wealth management capability.*

Cyber-risk self-protection is expected to moderate the relationship between trust in hybrid artificial intelligence advisory and digital wealth management behavior. In digital financial environments, investors may trust an AI-supported advisory system but still hesitate to act if they feel unable to protect their accounts, recognize fraudulent messages, or manage cyber-risk exposure. Protection Motivation Theory suggests that individuals are more likely to engage in protective or technology-related behaviors when they believe they can respond effectively to potential threats [13,14]. Information security research further shows that coping appraisal, including self-efficacy and response efficacy, plays an important role in motivating security-related behavior [16].

In hybrid AI advisory, cyber-risk self-protection may therefore serve as a boundary condition for converting trust into digital wealth-management behavior. Investors with stronger cyber-risk self-protection are likely to feel more capable of using digital platforms safely, which may make them more willing to act on trusted advisory recommendations. Conversely, investors with lower cyber-risk self-protection may remain cautious or inactive even when they trust the advisory system, because the perceived digital risk environment limits behavioral engagement. Thus, cyber-risk self-protection is conceptualized not merely as a direct security-related capability, but as a boundary condition that may help explain when trust becomes actionable.

***H10*** *: Cyber-risk self-protection positively moderates the relationship between trust in hybrid artificial intelligence advisory and digital wealth management behavior, such that the positive effect is stronger when cyber-risk self-protection is higher.*

Based on the hypothesized relationships, the proposed conceptual model integrating digital financial literacy, perceived AI advisory transparency, trust in hybrid AI advisory, investment and wealth knowledge, cyber-risk self-protection, and digital wealth management behavior is presented in Fig 1.

**Fig 1:**
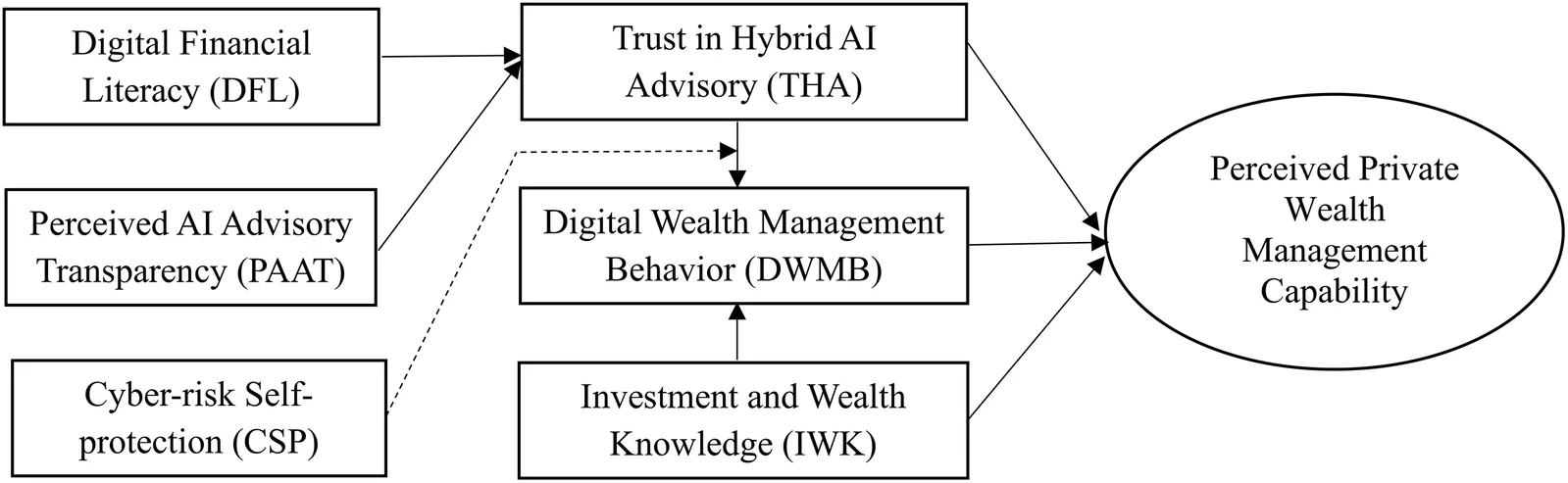
Proposed research model for digitally engaged retail investors. **Source:** Authors’ compilation.

## 2.5 Research model

## 3 Research methodology

### 3.1 Research design

This study employed a quantitative cross-sectional survey design to examine the determinants of perceived private wealth management capability among digitally engaged retail investors in Vietnam. The target respondents were individuals aged 18 years or older who met four screening conditions: (1) they had an active bank, securities, mutual fund, or digital investment account; (2) they had previously used a digital financial platform, investment application, robo-advisory tool, or AI-supported advisory function; (3) they had experience in monitoring, allocating, or adjusting personal financial assets; and (4) they reported having investable financial assets or recurring savings available for investment. These criteria were used to ensure that respondents had sufficient exposure to digital wealth management activities and could meaningfully evaluate hybrid AI advisory services. Respondents who did not satisfy all eligibility criteria were excluded from the survey.

A non-probability convenience sampling method was adopted because a complete sampling frame of Vietnamese investors who use digital financial services was unavailable. Accordingly, the findings should be interpreted within the surveyed sample rather than generalized as nationally representative estimates.

### 3.2 Measurement scales and questionnaire design

To enhance measurement transparency, the questionnaire adaptation process followed a structured procedure. Measurement items were adapted from established construct-specific scales reported in prior studies, as summarized in Table 1. The original conceptual definitions of each construct were retained, while wording was adjusted to reflect the hybrid AI-supported wealth management context. The adapted items were reviewed for contextual relevance, translated into Vietnamese, and compared item by item with the English version to ensure semantic consistency.

**Table 1:** Measurement sources and adaptation procedures.

Construct	Measurement source	Items	Adaptation procedure	Example item
Digital Financial Literacy (DFL)	Informed by [7,17]	3	Items were contextually adapted to assess investors’ ability to understand, evaluate, and use digital financial information in AI-supported wealth management environments.	I can evaluate financial information obtained from digital investment platforms before making decisions.
Perceived AI Advisory Transparency (PAAT)	Developed with reference to [12,18]	3	Items were refined to capture users’ perceptions of explainability, clarity, and understandability of AI-supported investment recommendations.	The AI advisory system provides explanations that help me understand its recommendations.
Trust in Hybrid AI Advisory (THA)	Adapted from trust-in-technology measures [25,26]	3	Trust-related items were adapted from general technology trust measures to reflect reliance on AI alongside human advisory support.	I believe the hybrid AI advisory system is reliable for supporting my investment decisions.
Investment and Wealth Knowledge (IWK)	Based on [24]	3	The original knowledge domain was operationalized as self-assessed knowledge of investment and wealth, suitable for a Likert-scale survey.	I understand the relationship between investment risk and expected return.
Digital Wealth Management Behavior (DWMB)	Informed by [19,27]	3	Behavioral items were adapted to represent digital portfolio monitoring, asset management activities, and the use of digital wealth tools.	I regularly monitor and adjust my investment portfolio using digital financial tools.
Cyber-risk Self-protection (CSP)	Adapted from [28,29]	3	Items were adjusted to reflect protective behaviors when using digital investment and AI-enabled financial services.	I take precautions to protect my financial accounts from online security threats.
Perceived Private Wealth Management Capability (PWMC)	Adapted from [22]	3	Items were refined to measure respondents’ perceived capability in managing personal wealth, rather than objective wealth outcomes.	I feel capable of managing my personal wealth effectively.

*Source: Authors’ compilation based on prior studies.*

The questionnaire was developed in English and Vietnamese. Before distribution, the two versions were compared item by item to ensure semantic consistency and contextual appropriateness. Following the eligibility screening, the questionnaire consisted of two sections. Before answering the main questionnaire, respondents completed screening questions to verify their eligibility. These questions corresponded to the four screening conditions described above: (1) an active bank, securities, mutual fund, or digital investment account; (2) previous use of a digital financial platform, investment application, robo-advisory tool, or AI-supported advisory function; (3) experience monitoring, allocating, or adjusting personal financial assets; and (4) investable financial assets or recurring savings available for investment. Only respondents who satisfied all screening conditions were allowed to proceed to the main survey. The first section collected demographic information, including gender, age, education, monthly income, investment experience, and risk tolerance. The second section measured the seven constructs in the proposed model.

All constructs were measured reflectively using 21 items, with three items assigned to each construct. Responses were recorded on a five-point Likert scale ranging from 1 = strongly disagree to 5 = strongly agree. Because the study relied on self-reported survey data, the constructs should be interpreted as perceived or self-assessed measures rather than objective performance indicators. Investment and wealth knowledge was operationalized as self-assessed investment and wealth knowledge, reflecting respondents’ perceived understanding of financial products, investment strategies, diversification, and risk-return trade-offs. Similarly, Perceived Private Wealth Management Capability (PWMC) captured respondents’ self-assessed ability to manage personal wealth, make informed decisions, and use knowledge and behavior to support financial outcomes. Therefore, the study does not claim to measure objective investment knowledge, actual portfolio performance, private-banking status, or verified wealth outcomes. The measurement items were adapted and contextualized from prior studies. Digital financial literacy was informed by [7,8,17,23]. Perceived artificial intelligence advisory transparency was developed with reference to [3,12,18]. The trust in hybrid artificial intelligence advisory was adapted from [25] and [26]. Investment and wealth knowledge were based on [24]. Digital wealth management behavior was informed by [27] and prior robo-advisory research on digital investment behavior and advisory engagement [19–21]. Cyber-risk self-protection was adapted from [28,29]. Perceived Private Wealth Management Capability was adapted from [22] for the context.

The cited source measures were selected because they correspond to the conceptual domains represented in the model: digital financial capability, perceived transparency and technology-specific trust, investment knowledge and financial behavior, cyber self-protection, and perceived financial capability. The items were contextually adapted to hybrid AI-supported wealth management while retaining the conceptual meaning of the source constructs; the English and Vietnamese versions were then compared item by item before pilot testing.

A pilot test was conducted with 10 Vietnamese individual investors who met the eligibility criteria. The pilot test confirmed the clarity, readability, and logical sequence of the questionnaire. No item was removed following this stage.

### 3.3 Data collection and research sample

Data were collected using Google Forms between 1 April 2026 and 30 May 2026. The online questionnaire was distributed across Vietnam through relevant investment communities and personal and professional networks. Before accessing the main questionnaire, participants were presented with an informed consent statement explaining the purpose of the study, the voluntary nature of participation, confidentiality safeguards, the right to withdraw at any time before submission, and the exclusive use of the data for academic research. Electronic informed consent was obtained by asking participants to confirm their agreement before proceeding to the questionnaire. Only individuals aged 18 years or older were eligible to participate; therefore, parental or guardian consent was not applicable. No names, email addresses, telephone numbers, IP addresses, or other personally identifiable information were collected.

Respondent eligibility was determined based on three criteria: (1) participants had experience with digital financial services or investment-related digital platforms, (2) they were familiar with AI-supported financial information or advisory features, and (3) they were capable of evaluating digital wealth-management decisions. Therefore, the sample represents digitally engaged retail investors rather than the broader population of Vietnamese investors.

The field procedure followed a sequential online process. Participants first reviewed the informed-consent statement, then completed the eligibility screening questions, and only eligible respondents proceeded to the demographic and construct measures. After the collection period, responses were screened for completeness; 19 incomplete submissions were excluded, leaving 249 complete observations for analysis.

Ethical approval was granted by the Research Ethics Committee of Ho Chi Minh City University of Economics and Finance (Approval No. 402/QD-UEF, dated 27 March 2026). The Principal Investigator attended the ethics review meeting solely to present the research proposal and respond to questions; the ethical approval decision was made independently by the voting members of the Ethics Review Council.

A total of 268 responses were initially collected. After excluding 19 incomplete responses, 249 complete and usable observations were retained for analysis. Although the study targeted digitally engaged retail investors with exposure to investment platforms and AI-supported advisory services, the final sample should be interpreted as a sample of digitally engaged retail investors rather than a nationally representative sample of all investors, private banking clients, or high-net-worth individuals. To assess sample adequacy, a sensitivity-based statistical power analysis was conducted using G*Power 3.1 [30]. The most complex endogenous construct in the proposed model was digital wealth management behavior, which was predicted by four terms: trust in hybrid artificial intelligence advisory, investment and wealth knowledge, cyber-risk self-protection, and the interaction term between cyber-risk self-protection and trust in hybrid artificial intelligence advisory. Following the statistical power approach recommended for PLS-SEM research [31], the analysis was conducted at a significance level of 0.05, a power level of 0.80, with four predictors, and a conservative minimum effect size of f^2^ = 0.05. The resulting minimum required sample size was 244 observations. The final sample of 249 valid responses, therefore, exceeded this requirement and was considered adequate for estimating the proposed structural model.

### 3.4 Quantitative data analysis

Microsoft Excel and IBM SPSS Statistics were used for data screening, coding verification, and descriptive analysis. The measurement and structural models were assessed using partial least squares structural equation modeling in SmartPLS 4.1. PLS-SEM was selected because the study is prediction-oriented and incorporates multiple latent constructs, two mediating relationships, and one moderating effect [31,32].

The reflective measurement model was evaluated using outer loadings, Cronbach’s alpha, rho_A, composite reliability, and average variance extracted. Discriminant validity was assessed primarily through the heterotrait–monotrait ratio of correlations and bootstrap confidence intervals, while the Fornell–Larcker criterion was reported as a supplementary diagnostic [33,34]. Collinearity was examined using variance inflation factor values.

The structural model was assessed using path coefficients, t-values, p-values, and bias-corrected 95% confidence intervals based on 5,000 bootstrap subsamples. Explanatory power and effect sizes were evaluated using R^2^, adjusted R^2^, and f^2^ values. Specific indirect effects were examined to test mediation, while the interaction term between cyber-risk self-protection and trust in hybrid artificial intelligence advisory was used to evaluate moderation.

Potential common method bias was assessed using both Harman’s single-factor test and the full collinearity variance inflation factor approach proposed by [35]. The corresponding results are reported in Section 4.2.4. Finally, out-of-sample predictive performance was examined using PLSpredict with 10 folds, 10 repetitions, and a fixed seed [36]. The standardized root mean square residual was reported as a supplementary approximate model-fit diagnostic rather than as a definitive global goodness-of-fit index.

To further interpret the moderating effect, the interaction was probed using a simple slope analysis at low and high levels of cyber-risk self-protection. Low and high levels were operationalized as one standard deviation below and above the mean, respectively. This additional analysis was used to assess whether the positive effect of trust in hybrid AI advisory on digital wealth management behavior differed meaningfully across different levels of cyber-risk self-protection.

## 4 PLS-SEM research results

### 4.1 Descriptive statistics of the sample

Table 2 summarizes the demographic profile of the final sample. The final sample consisted of 249 valid responses. Regarding gender, female respondents accounted for 59.8% of the sample, while male respondents represented 40.2%. By age, the largest group was respondents aged 30–39, at 39.0%, followed by those aged 18–29 at 32.9% and those aged 40–45 at 23.3%. Respondents over 45 years old represented only 4.8% of the sample. This indicates that the sample was mainly composed of young and middle-aged investors, which is relevant to the context of digital financial services and investment behavior.

**Table 2:** Descriptive statistics of the sample.

Variable	Category	Frequency	Percentage
Gender	Male	100	40.2%
	Female	149	59.8%
Age	18–29	82	32.9%
	30–39	97	39.0%
	40–45	58	23.3%
	Over 45	12	4.8%
Education	Bachelor	94	37.8%
	Postgraduate	74	29.7%
	College/Intermediate	66	26.5%
	High School	15	6.0%
	Other	0	0.0%
Income	<$200	0	0.0%
	$200–$400	19	7.6%
	$400–$800	76	30.5%
	$800–$1,000	112	45.0%
	> $1,000	42	16.9%
Investment Experience	<1 year	0	0.0%
	1–3 years	24	9.6%
	3–5 years	46	18.5%
	5–7 years	96	38.6%
	>7 years	83	33.3%
Risk Tolerance	Very Low	81	32.5%
	Low	69	27.7%
	Medium	38	15.3%
	High	36	14.5%
	Very High	25	10.0%
Total valid observations		**249**	**100.0**

Note: *Percentages are calculated using 249 complete observations. All respondents included in the final sample were aged 18 years or older and satisfied all four screening conditions by confirming: (1) an active bank, securities, mutual fund, or digital investment account; (2) previous use of a digital financial platform, investment application, robo-advisory tool, or AI-supported advisory function; (3) experience monitoring, allocating, or adjusting personal financial assets; and (4) investable financial assets or recurring savings available for investment. Respondents who did not meet these eligibility criteria were automatically excluded before entering the main questionnaire.*

*Source: Authors’ calculations based on the survey dataset.*

Regarding education, most respondents had relatively high levels of schooling. Bachelor’s degree holders formed the largest group, accounting for 37.8% of the sample, followed by postgraduate respondents at 29.7% and college/intermediate graduates at 26.5%. Respondents with a high school education accounted for 6.0%, while no respondents selected the “Other” category. This distribution suggests that the respondents generally had sufficient educational backgrounds to understand investment information and answer questions related to financial decision-making.

In terms of monthly income, the largest proportion of respondents reported earning $800–$1,000, accounting for 45.0% of the sample. Respondents earning $400–$800 represented 30.5%, while those earning more than $1,000 accounted for 16.9%. Only 7.6% of respondents reported income between $200 and $400, and no respondents reported income below $200. This shows that the sample was concentrated among individuals with moderate to relatively high income levels, which is appropriate for a study examining investment-related behavior.

Regarding investment experience, most respondents had considerable investment experience. The largest group had 5–7 years of investment experience, accounting for 38.6%, followed by those with more than 7 years of experience at 33.3%. Respondents with 3–5 years of experience represented 18.5%, while those with 1–3 years accounted for 9.6%. No respondents had less than one year of investment experience. These findings indicate that the sample mainly consisted of experienced investors, which strengthens the relevance of their responses to the study context.

Regarding risk tolerance, the largest group reported very low tolerance (32.5%), followed by low tolerance (27.7%). Respondents with medium risk tolerance represented 15.3%, while those with high and very high risk tolerance accounted for 14.5% and 10.0%, respectively. This pattern suggests that although the sample had relatively high investment experience, many respondents remained cautious in their investment decisions.

### 4.2 Measurement model assessment

#### 4.2.1 Reliability, internal consistency, convergent validity, and indicator collinearity.

Table 3 reports the reflective measurement model assessment. The reflective measurement model was evaluated by examining indicator reliability, internal consistency, convergent validity, and indicator-level multicollinearity. Following [32], satisfactory indicator reliability is established when outer loadings exceed 0.708, while internal consistency reliability and convergent validity require reliability coefficients above 0.70 and average variance extracted values above 0.50.

**Table 3:** Reflective measurement model assessment.

Symbol	Item	Outer Loading	Cronbach Alpha	rho_a	rho_c	AVE	VIF
**Digital Financial Literacy (DFL)**							
DFL1	I can evaluate digital financial products effectively.	0.920	0.910	0.911	0.944	0.848	3.082
DFL2	I understand how to use online investment platforms.	0.922					3.073
DFL3	I can critically assess digital financial information.	0.920					3.007
**Perceived AI Advisory Transparency (PAAT)**							
PAAT1	AI recommendations are clearly explained.	0.879	0.830	0.837	0.898	0.746	1.933
PAAT2	I understand how AI generates financial suggestions.	0.849					1.902
PAAT3	The AI advisory process is transparent and understandable.	0.863					1.875
**Trust in Hybrid AI Advisory (THA)**							
THA1	I trust that AI + human advisory provides reliable recommendations.	0.879	0.834	0.835	0.900	0.750	2.000
THA2	I believe the hybrid advisory system is competent.	0.851					1.833
THA3	I feel confident relying on the hybrid AI advisory.	0.869					1.978
**Investment and Wealth Knowledge (IWK)**							
IWK1	I understand financial products and investment strategies.	0.890	0.878	0.879	0.925	0.804	2.314
IWK2	I can evaluate investment opportunities effectively.	0.895					2.425
IWK3	I am knowledgeable about risk-return trade-offs.	0.905					2.487
**Digital Wealth Management Behavior (DWMB)**							
DWMB1	I regularly monitor and adjust my digital investment portfolio.	0.940	0.918	0.921	0.948	0.859	3.739
DWMB2	I consistently consider AI and human advisory recommendations.	0.912					2.969
DWMB3	I use digital tools to manage my wealth proactively.	0.927					3.355
**Cyber-risk Self-Protection (CSP)**							
CSP1	I take precautions to secure my online financial accounts.	0.846	0.843	0.843	0.905	0.761	1.806
CSP2	I can recognize potential online financial threats.	0.864					2.088
CSP3	I actively prevent cybersecurity risks when using financial platforms.	0.906					2.562
**Perceived Private Wealth Management Capability (PWMC)**							
PWMC1	I am capable of managing my personal wealth effectively.	0.905	0.872	0.873	0.922	0.797	2.559
PWMC2	I can optimize financial outcomes using knowledge and behavior.	0.870					2.057
PWMC3	My wealth management decisions are informed and consistent.	0.902					2.536

Note: DFL: Digital Financial Literacy, PAAT: Perceived AI Advisory Transparency, THA: Trust in Hybrid AI Advisory, DWMB: Digital Wealth Management Behavior, IWK: Investment and Wealth Knowledge, PWMC: Perceived Private Wealth Management Capability, CSP: Cyber-risk Self-protection.

*Source: Authors’ calculations using SmartPLS 4.1.*

The outer loadings range from 0.846 to 0.940. All indicators exceeded the recommended threshold of 0.708 and were therefore retained. The highest loading is observed for the first indicator of digital wealth management behavior (0.940), whereas the lowest loading is reported for the first indicator of cyber-risk self-protection (0.846). These results demonstrate satisfactory indicator reliability across all reflective constructs.

Internal consistency reliability is also established. Cronbach’s alpha values range from 0.830 to 0.918, rho_A values range from 0.835 to 0.921, and composite reliability values range from 0.898 to 0.948. All values exceed the minimum threshold of 0.70 and remain below the upper benchmark of 0.95. The constructs, therefore, demonstrate satisfactory reliability, with no strong evidence of excessive item redundancy.

Convergent validity is supported because the average variance extracted values range from 0.746 to 0.858, exceeding the recommended threshold of 0.50. Specifically, the average variance extracted values are 0.848 for digital financial literacy, 0.746 for perceived artificial intelligence advisory transparency, 0.751 for trust in hybrid artificial intelligence advisory, 0.804 for investment and wealth knowledge, 0.858 for digital wealth management behavior, 0.761 for cyber-risk self-protection, and 0.797 for private wealth management capability. These results confirm that each construct explains more than half of the variance in its corresponding indicators.

The outer variance inflation factor values range from 1.806 to 3.739. All values remain below the critical threshold of 5.00, indicating the absence of severe indicator-level collinearity. Although selected indicators of digital financial literacy and digital wealth management behavior are slightly above the conservative benchmark of 3.00, they remain acceptable and do not warrant indicator removal.

#### 4.2.2 Discriminant validity assessment using the HTMT criterion.

Table 4 reports the HTMT assessment. Discriminant validity was assessed using the heterotrait–monotrait ratio of correlations [34]. Henseler et al. [34] recommend the heterotrait–monotrait ratio as a more sensitive criterion than the Fornell–Larcker criterion and cross-loading inspection for detecting discriminant validity issues in PLS-SEM. The heterotrait–monotrait values among the substantive constructs range from 0.563 to 0.743. The highest value is observed between digital financial literacy and trust in hybrid artificial intelligence advisory (0.743), followed by the relationship between cyber-risk self-protection and digital wealth management behavior (0.742), and the relationship between investment and wealth knowledge and digital wealth management behavior (0.735). All values remain below the conservative threshold of 0.85. Therefore, the constructs demonstrate satisfactory discriminant validity.

**Table 4:** Discriminant validity assessment using the HTMT criterion.

	CSP	DFL	DWMB	IWK	PAAT	PWMC	THA	CSP x THA
**CSP**								
**DFL**	0.661							
**DWMB**	0.742	0.702						
**IWK**	0.625	0.675	0.735					
**PAAT**	0.707	0.577	0.617	0.563				
**PWMC**	0.630	0.708	0.680	0.630	0.594			
**THA**	0.683	0.743	0.703	0.638	0.622	0.641		
**CSP x THA**	0.035	0.042	0.062	0.034	0.112	0.105	0.134	

Note: DFL: Digital Financial Literacy, PAAT: Perceived AI Advisory Transparency, THA: Trust in Hybrid AI Advisory, DWMB: Digital Wealth Management Behavior, IWK: Investment and Wealth Knowledge, PWMC: Perceived Private Wealth Management Capability, CSP: Cyber-risk Self-protection.

*Source: Authors’ calculations using SmartPLS 4.1.*

The interaction term between cyber-risk self-protection and trust in hybrid artificial intelligence advisory exhibits heterotrait–monotrait values ranging from 0.034 to 0.134. As the interaction term was introduced specifically to test the moderating effect, these values are reported for completeness but are not interpreted as evidence relating to a standalone theoretical construct.

Taken together, the results indicate that the constructs capture empirically distinct dimensions of the proposed model. The discriminant validity of the reflective measurement model is therefore established.

To further verify discriminant validity, HTMT inference was assessed using bootstrapped confidence intervals. The upper bounds of the 95% confidence intervals range from 0.678 to 0.823, all below the conservative threshold of 0.85. The highest upper bound is observed for the relationship between digital financial literacy and trust in hybrid artificial intelligence advisory (0.823). These findings provide further evidence that discriminant validity is established across all substantive constructs.

#### 4.2.3 Discriminant validity assessment using the Fornell-Larcker criterion.

Table 5 presents the supplementary Fornell–Larcker assessment. The Fornell–Larcker criterion was examined as a supplementary assessment of discriminant validity. According to [33], discriminant validity is supported when the square root of the average variance extracted for each construct exceeds its correlations with all other constructs in the model. The diagonal values range from 0.864 to 0.927 and exceed the corresponding inter-construct correlations in all cases. For example, the square root of the average variance extracted for digital financial literacy is 0.921, which is higher than its strongest correlation with trust in hybrid artificial intelligence advisory (0.648). Similarly, the square root of the average variance extracted for digital wealth management behavior is 0.927, exceeding its highest correlation with investment and wealth knowledge (0.661). The square root of the average variance extracted for trust in hybrid artificial intelligence advisory is 0.866, which also exceeds its correlations with the other constructs.

**Table 5:** Discriminant validity assessment using the Fornell–Larcker criterion.

	CSP	DFL	DWMB	IWK	PAAT	PWMC	THA
**CSP**	0.872						
**DFL**	0.579	0.921					
**DWMB**	0.652	0.643	0.927				
**IWK**	0.537	0.603	0.661	0.896			
**PAAT**	0.593	0.502	0.539	0.481	0.864		
**PWMC**	0.540	0.632	0.611	0.551	0.507	0.893	
**THA**	0.573	0.648	0.615	0.545	0.521	0.548	0.866

Note: DFL: Digital Financial Literacy, PAAT: Perceived AI Advisory Transparency, THA: Trust in Hybrid AI Advisory, DWMB: Digital Wealth Management Behavior, IWK: Investment and Wealth Knowledge, PWMC: Perceived Private Wealth Management Capability, CSP: Cyber-risk Self-protection.

*Source: Authors’ calculations using SmartPLS 4.1.*

These results provide supplementary evidence that the constructs are empirically distinct. Combined with the heterotrait–monotrait ratio results and the bootstrapped confidence intervals, the findings confirm satisfactory discriminant validity across the measurement model.

#### 4.2.4 Common method bias assessment.

Because this study relied on cross-sectional self-reported survey data, common method bias was assessed before interpreting the structural model. Common method bias may occur when the observed relationships among constructs are partly attributable to the measurement method rather than to the substantive theoretical relationships being examined [37,38]. To address this concern procedurally, the questionnaire informed respondents about confidentiality, voluntary participation, and the study’s academic purpose, and no personally identifiable information was collected. However, because all constructs were measured using the same self-reported survey instrument, statistical diagnostics were still necessary.

Table 6 reports the Harman single-factor results. First, Harman’s single-factor test was conducted using principal component analysis with an unrotated single-factor solution. The first factor had an eigenvalue of 10.673 and explained 50.823% of the total variance across the 21 measurement items. This result slightly exceeds the commonly applied 50% benchmark and therefore suggests a borderline concern. Since Harman’s single-factor test has been criticized for its limited sensitivity in detecting common method bias, this result should not be used alone to conclude that method bias is absent [37,39].

**Table 6:** Common method bias assessment using harman’s single-factor test.

Test	Extraction method	Number of items	First factor eigenvalue	Variance explained by the first factor
Harman’s single-factor test	Principal Component Analysis, unrotated	21	10.673	50.823%

*Source: Authors’ calculations using SPSS 27*

Table 7 reports the full-collinearity VIF results. Second, the full collinearity VIF approach was applied as an additional diagnostic procedure. This approach is recommended in PLS-SEM research for detecting potential common method bias by examining whether latent constructs are excessively explained by the remaining constructs in the model [35]. The full collinearity VIF values ranged from 1.761 to 2.646, which are below the conservative threshold of 3.3 suggested by [35]. These results indicate that severe common method bias is unlikely to dominate the estimated relationships.

**Table 7:** Full collinearity VIF.

Construct	R^2^	Full collinearity VIF
DFL	0.587	2.421
THA	0.524	2.101
PWMC	0.503	2.012
PAAT	0.432	1.761
CSP	0.540	2.174
DWMB	0.622	2.646
IWK	0.512	2.049

*Source: Authors’ calculations using SPSS 27*

Taken together, the findings should be interpreted with appropriate caution. Harman’s single-factor test indicates a borderline result, whereas the full collinearity VIF results provide stronger evidence that common method bias is unlikely to distort the structural model materially. Therefore, common method bias does not appear to pose a serious threat to the findings, although it cannot be completely ruled out given the cross-sectional and self-reported nature of the data.

#### 4.2.5 Structural collinearity assessment using inner VIF.

Table 8 reports the inner VIF values for the structural model. Before assessing the structural relationships, collinearity among the predictor constructs was examined using the inner variance inflation factor values [32]. Recommend evaluating the variance inflation factor values of each set of predictor constructs before interpreting path coefficients, explanatory power, and effect sizes. The inner variance inflation factor values range from 1.026 to 2.125. Digital financial literacy and perceived artificial intelligence advisory transparency, which jointly predict trust in hybrid artificial intelligence advisory, both exhibit variance inflation factor values of 1.337. These results indicate that the two antecedents capture distinct user-related and system-related dimensions without creating problematic collinearity.

**Table 8:** Inner VIF values for the structural model.

	VIF
**CSP - > DWMB**	1.668
**CSP x THA - > DWMB**	1.026
**DFL - > THA**	1.337
**DWMB - > PWMC**	2.125
**IWK - > DWMB**	1.607
**IWK - > PWMC**	1.879
**PAAT - > THA**	1.337
**THA - > DWMB**	1.727
**THA - > PWMC**	1.703

Note: DFL: Digital Financial Literacy, PAAT: Perceived AI Advisory Transparency, THA: Trust in Hybrid AI Advisory, DWMB: Digital Wealth Management Behavior, IWK: Investment and Wealth Knowledge, PWMC: Perceived Private Wealth Management Capability, CSP: Cyber-risk Self-protection.

*Source: Authors’ calculations using SmartPLS 4.1.*

For digital wealth management behavior, the variance inflation factor values are 1.668 for cyber-risk self-protection, 1.607 for investment and wealth knowledge, 1.727 for trust in hybrid artificial intelligence advisory, and 1.026 for the interaction term between cyber-risk self-protection and trust in hybrid artificial intelligence advisory. The low value associated with the interaction term indicates that the moderation specification does not introduce substantial collinearity into the structural model.

Finally, the variance inflation factor values for the predictors of perceived private wealth management capability are 2.125 for digital wealth management behavior, 1.879 for investment and wealth knowledge, and 1.703 for trust in hybrid artificial intelligence advisory. The highest value in the structural model is therefore 2.125, which remains below both the conservative threshold of 3.00 and the commonly applied threshold of 5.00.

### 4.3 Structural model assessment

#### 4.3.1 Coefficient of determination (R^2^).

Table 9 summarizes R^2^ and adjusted R^2^ for the endogenous constructs. The explanatory power of the structural model was assessed using the coefficient of determination. The coefficient of determination represents the proportion of variance explained in each endogenous construct and should be interpreted in relation to the research context and the complexity of the model [31,32]. As general reference points, values of 0.75, 0.50, and 0.25 may be considered substantial, moderate, and weak, respectively. Digital financial literacy and perceived transparency of artificial intelligence advisory explain 47.1% of the variance in trust in hybrid artificial intelligence advisory. The corresponding adjusted coefficient of determination is 0.467, indicating a meaningful level of explanatory power approaching the moderate benchmark. The predictors of digital wealth management behavior explain 60.2% of its variance, with an adjusted coefficient of determination of 0.595. This result indicates moderate explanatory power and suggests that trust in hybrid artificial intelligence advisory, investment, and wealth knowledge, cyber-risk self-protection, and the interaction term jointly provide a substantive explanation of individuals’ digital wealth management behavior. Finally, trust in hybrid artificial intelligence advisory, digital wealth management behavior, and investment and wealth knowledge explain 44.3% of the variance in perceived private wealth management capability. The adjusted coefficient of determination is 0.436. Although this value remains below the moderate benchmark of 0.50, it demonstrates meaningful weak-to-moderate explanatory power in the context of individual wealth management, where additional personal, financial, and market-related factors beyond the scope of the present model may also shape perceived capability.

**Table 9:** R^2^ and adjusted R^2^ values of the endogenous constructs.

	R-square	R-square adjusted
**THA**	0.471	0.467
**DWMB**	0.602	0.595
**PWMC**	0.443	0.436

Note: THA: Trust in Hybrid AI Advisory, DWMB: Digital Wealth Management Behavior, PWMC: Perceived Private Wealth Management Capability.

*Source: Authors’ calculations using SmartPLS 4.1.*

The small differences between the coefficient of determination and the adjusted coefficient of determination values, ranging from 0.004 to 0.007, indicate that its predictor specification does not materially inflate the model’s explanatory power. Nevertheless, the model’s out-of-sample predictive performance is examined separately using PLSpredict.

#### 4.3.2 Effect size assessment (f^2^).

In addition to the coefficient of determination, the effect size was examined to evaluate the contribution of each predictor construct to the explanatory power of the endogenous constructs. Following [32], values of 0.02, 0.15, and 0.35 indicate small, moderate, and large effects, respectively. As shown in Table 10, digital financial literacy has a large effect on trust in hybrid artificial intelligence advisory (f^2^ = 0.379), representing the strongest contribution in the structural model. Perceived transparency of artificial intelligence advisory has a small effect on trust in hybrid artificial intelligence advisory (f^2^ = 0.096). These findings suggest that advisory transparency contributes to trust formation. At the same time, individuals’ ability to evaluate digital financial information plays a more substantial role in developing informed reliance on hybrid advisory systems.

**Table 10:** Effect size (f^2^) matrix.

	CSP	DFL	DWMB	IWK	PAAT	PWMC	THA	CSP x THA
**CSP**			0.157					
**DFL**							0.379	
**DWMB**						0.093		
**IWK**			0.189			0.040		
**PAAT**							0.096	
**PWMC**								
**THA**			0.092			0.057		
**CSP x THA**			0.022					

Note: DFL: Digital Financial Literacy, PAAT: Perceived AI Advisory Transparency, THA: Trust in Hybrid AI Advisory, DWMB: Digital Wealth Management Behavior, IWK: Investment and Wealth Knowledge, PWMC: Perceived Private Wealth Management Capability, CSP: Cyber-risk Self-protection, CSP × THA: interaction term between Cyber-risk Self-protection and Trust in Hybrid AI Advisory

*Source: Authors’ calculations using SmartPLS 4.1.*

For digital wealth management behavior, investment and wealth knowledge have a moderate effect (f^2^ = 0.189), followed by cyber-risk self-protection (f^2^ = 0.157). Trust in hybrid artificial intelligence advisory has a small effect on digital wealth management behavior (f^2^ = 0.092). The interaction term between cyber-risk self-protection and trust in hybrid artificial intelligence advisory also has a small effect (f^2^ = 0.022). Although this interaction is statistically significant, its limited magnitude indicates that cyber-risk self-protection should be interpreted as a modest boundary condition rather than as a dominant explanatory mechanism.

Regarding perceived private wealth management capability, digital wealth management behavior has the largest effect among the three predictors, although the magnitude remains small (f^2^ = 0.093). Trust in hybrid artificial intelligence advisory (f^2^ = 0.057) and investment and wealth knowledge (f^2^ = 0.040) also exhibit small effects. These findings suggest that perceived private wealth management capability is shaped by complementary mechanisms involving trust, knowledge, and enacted digital behavior rather than by a single dominant predictor.

#### 4.3.3 Hypothesis testing results.

Table 11 reports the direct, indirect, and moderating effects. The statistical significance of the proposed relationships was evaluated using the bootstrapping procedure. Following [31,32], a relationship was considered statistically significant when its p-value was below 0.05 and the corresponding bias-corrected 95% bootstrap confidence interval excluded zero. All proposed hypotheses were supported. Digital financial literacy has a significant positive effect on trust in hybrid artificial intelligence advisory (β = 0.517, t = 9.326, p < 0.001, 95% CI [0.402, 0.623]), supporting H1. This is the strongest direct relationship in the model. Perceived transparency of artificial intelligence advisory also positively influences trust in hybrid artificial intelligence advisory (β = 0.261, t = 4.241, p < 0.001, 95% CI [0.142, 0.380]), supporting H2. These findings indicate that trust is shaped by both users’ digital financial competence and their perceptions of system transparency.

**Table 11:** Results of direct, indirect, and moderating effects.

Hypothesis - Path/Relationship		Original sample (O)	Sample mean (M)	Standard deviation (STDEV)	T statistics (|O/STDEV|)	P values	95% CI LL	95% CI UL
**Direct effect**								
H1	**DFL -> THA**	0.517	0.517	0.055	9.326	< 0.001	0.402	0.623
H2	**PAAT -> THA**	0.261	0.262	0.061	4.241	< 0.001	0.142	0.380
H3	**THA -> DWMB**	0.252	0.251	0.063	3.970	< 0.001	0.132	0.381
H4	**IWK -> DWMB**	0.348	0.349	0.057	6.092	< 0.001	0.236	0.457
H5	**THA -> PWMC**	0.232	0.231	0.086	2.699	0.007	0.059	0.395
H6	**DWMB -> PWMC**	0.332	0.332	0.085	3.899	< 0.001	0.162	0.496
H7	**IWK -> PWMC**	0.205	0.208	0.074	2.756	0.006	0.055	0.349
**Mediating effect**								
H8	**THA -> DWMB -> PWMC**	0.084	0.084	0.032	2.641	0.008	0.036	0.165
H9	**IWK -> DWMB -> PWMC**	0.116	0.114	0.031	3.719	< 0.001	0.062	0.186
**Moderating effect**								
H10	**CSP x THA -> DWMB**	0.098	0.092	0.048	2.070	0.038	0.001	0.183

*Note: DFL: Digital Financial Literacy, PAAT: Perceived AI Advisory Transparency, THA: Trust in Hybrid AI Advisory, DWMB: Digital Wealth Management Behavior, IWK: Investment and Wealth Knowledge, PWMC: Perceived Private Wealth Management Capability, CSP: Cyber-risk Self-protection. CI values are bias-corrected bootstrap 95% confidence intervals; LL = lower limit; UL = upper limit.*

*Source: Authors’ calculations using SmartPLS 4.1.*

Trust in hybrid artificial intelligence advisory positively affects digital wealth management behavior (β = 0.252, t = 3.970, p < 0.001, 95% CI [0.132, 0.381]), supporting H3. Investment and wealth knowledge also has a significant positive effect on digital wealth management behavior (β = 0.348, t = 6.092, p < 0.001, 95% CI [0.236, 0.457]), supporting H4. The latter relationship is stronger, suggesting that investment-related knowledge plays a particularly important role in promoting active digital wealth management practices.

Regarding perceived private wealth management capability, trust in hybrid artificial intelligence advisory exerts a significant positive direct effect (β = 0.232, t = 2.699, p = 0.007, 95% CI [0.059, 0.395]), supporting H5. Digital wealth management behavior also positively influences perceived private wealth management capability (β = 0.332, t = 3.899, p < 0.001, 95% CI [0.162, 0.496]), supporting H6. In addition, investment and wealth knowledge have a significant direct effect on perceived private wealth management capability (β = 0.205, t = 2.756, p = 0.006, 95% CI [0.055, 0.349]), supporting H7.

The mediation analysis further indicates that digital wealth management behavior significantly transmits the influence of trust in hybrid artificial intelligence advisory to perceived private wealth management capability (β = 0.084, t = 2.641, p = 0.008, 95% CI [0.036, 0.165]), supporting H8. As the corresponding direct effect remains positive and significant, the result indicates complementary partial mediation. Digital wealth management behavior also mediates the relationship between investment and wealth knowledge and perceived private wealth management capability (β = 0.116, t = 3.719, p < 0.001, 95% CI [0.062, 0.186]), supporting H9. The significant and positive direct effect similarly indicates complementary partial mediation.

The interaction term between cyber-risk self-protection and trust in hybrid artificial intelligence advisory has a positive effect on digital wealth management behavior (β = 0.098, t = 2.070, p = 0.038, 95% CI [0.001, 0.183]), supporting H10. This result indicates that cyber-risk self-protection provides modest support as a boundary condition in the trust-to-behavior relationship. Nevertheless, the magnitude of the moderating effect is small and should be interpreted cautiously.

#### 4.3.4 Moderation and simple slope analysis.

Although the direct path from cyber-risk self-protection to digital wealth management behavior was not specified as a separate hypothesis, it was estimated as a constituent effect required for testing the moderating relationship between cyber-risk self-protection and trust in hybrid AI advisory. In moderation analysis, the lower-order effects of the interacting constructs should be included together with the interaction term to avoid misspecification and to ensure that the moderating effect is interpreted correctly [32,40]. Therefore, the CSP → DWMB path is reported for statistical completeness but is not treated as an independent theoretical hypothesis.

Table 12 reports the non-hypothesized constituent effect included in the moderation model. The result shows that cyber-risk self-protection has a significant positive constituent effect on digital wealth management behavior (β = 0.323, t = 5.100, p < 0.001, 95% CI [0.190, 0.439]). This indicates that respondents with stronger cyber-risk self-protection tend to engage more actively in digital wealth management behavior. However, because this path was included primarily to support the estimation of the interaction model, the substantive interpretation remains focused on the hypothesized moderating effect of CSP × THA on DWMB.

**Table 12:** Non-hypothesized constituent effect in the moderation model.

Path	Original sample (O)	Sample mean (M)	Standard deviation (STDEV)	T statistics (|O/STDEV|)	P values	95% CI LL	95% CI UL
*CSP → DWMB*	0.323	0.325	0.063	5.100	< 0.001	0.190	0.439

*Note: DWMB: Digital Wealth Management Behavior, CSP: Cyber-risk Self-protection. CI values are bias-corrected bootstrap 95% confidence intervals; LL = lower limit; UL = upper limit.*

*Source: Authors’ calculations using SmartPLS 4.1.*

The interaction term between cyber-risk self-protection and trust in hybrid artificial intelligence advisory has a positive and statistically significant effect on digital wealth management behavior (β = 0.098, t = 2.070, p = 0.038, 95% CI [0.001, 0.183]), supporting H10. However, the confidence interval is close to zero, and the effect size is small (f^2^ = 0.022). Therefore, the moderating effect should be interpreted cautiously as a statistically significant but substantively modest boundary condition rather than as a strong moderating mechanism.

Table 13 presents the simple-slope results. To further interpret the interaction, a simple slope analysis was conducted at low and high levels of cyber-risk self-protection. The conditional effect of trust in hybrid AI advisory on digital wealth management behavior was weaker when cyber-risk self-protection was low (−1 SD; β = 0.154) and stronger when cyber-risk self-protection was high (+1 SD; β = 0.350). This pattern is consistent with the hypothesized moderation effect, suggesting that trust in hybrid AI advisory is more likely to translate into digital wealth management behavior when investors perceive themselves as more capable of managing cyber risks. Nevertheless, given the small interaction effect and the narrow confidence interval near zero, this result should be interpreted as modest, supportive evidence for a boundary condition.

**Table 13:** Simple slope analysis of the moderating effect.

Level of cyber-risk self-protection	Conditional effect of THA on DWMB	Interpretation
Low CSP (−1 SD)	0.154	Positive but weaker
High CSP (+1 SD)	0.350	Positive and stronger

*Note: THA = Trust in Hybrid AI Advisory; DWMB = Digital Wealth Management Behavior; CSP = Cyber-risk Self-protection. Conditional effects were calculated based on the standardized coefficient of THA → DWMB and the interaction coefficient of CSP × THA → DWMB.*

*Source: Authors’ calculations using SmartPLS 4.1.*

#### 4.3.5 Structural model results.

Fig 2 presents the estimated structural model and standardized path coefficients.

**Fig 2:**
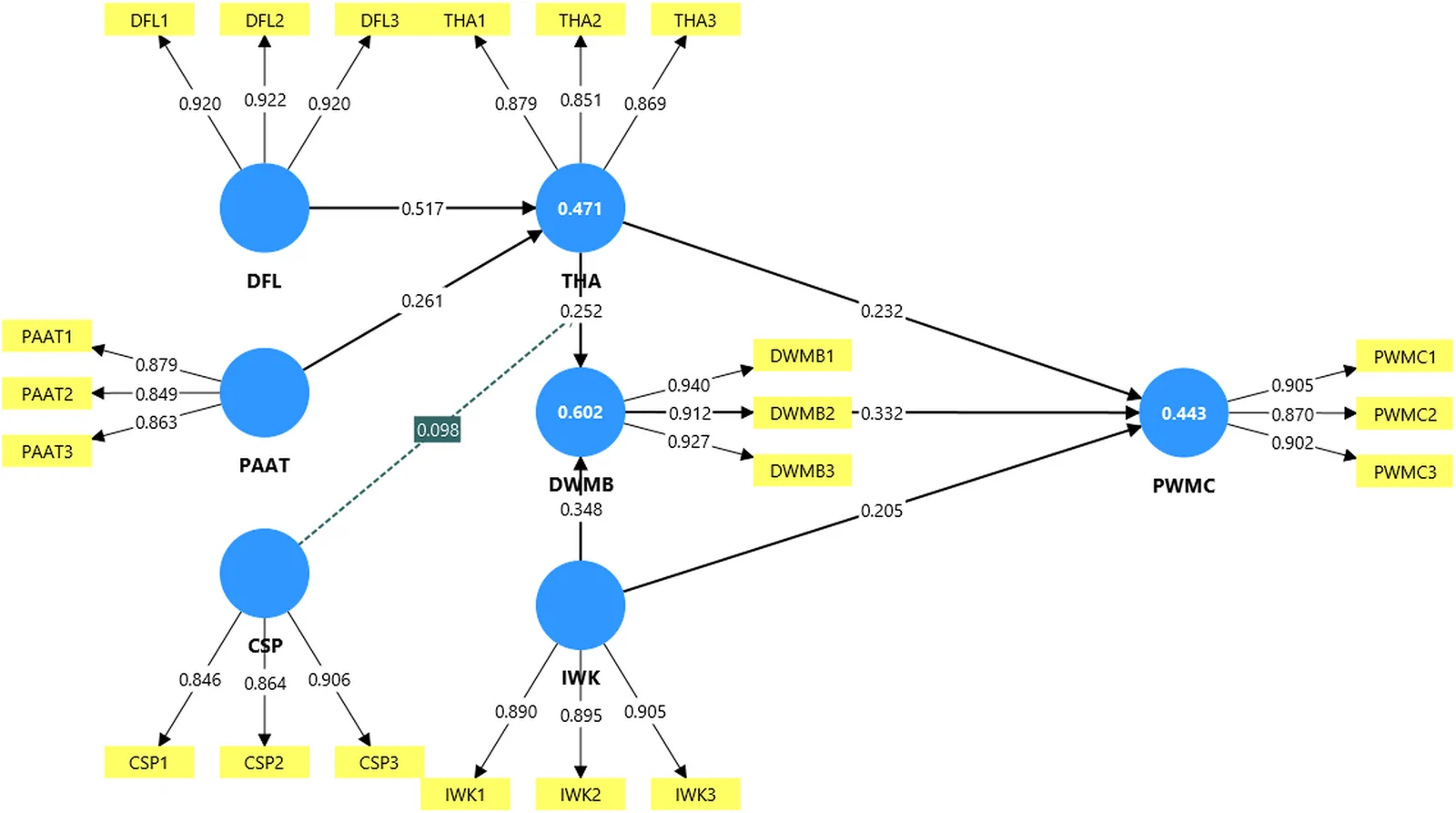
Estimated structural model for digitally engaged retail investors. Note: DFL: Digital Financial Literacy, PAAT: Perceived AI Advisory Transparency, THA: Trust in Hybrid AI Advisory, DWMB: Digital Wealth Management Behavior, IWK: Investment and Wealth Knowledge, PWMC: Perceived Private Wealth Management Capability, CSP: Cyber-risk Self-protection. *Source: Authors’ calculations using SmartPLS 4.1.*

The structural relationships proposed in the research model were assessed using the bootstrapping procedure in SmartPLS 4.1 with 5,000 subsamples. Following the PLS-SEM guidelines, a structural path was considered statistically significant when the p-value was below 0.05, and the corresponding bias-corrected 95% bootstrap confidence interval did not include zero [31,32]. The results provide support for all hypothesized direct, mediating, and moderating relationships.

For trust formation, digital financial literacy exerted a strong positive effect on trust in hybrid AI advisory (β = 0.517, t = 9.326, p < 0.001, 95% CI [0.402, 0.623]), supporting H1. Perceived AI advisory transparency also had a significant positive effect on trust in hybrid AI advisory (β = 0.261, t = 4.241, p < 0.001, 95% CI [0.142, 0.380]), supporting H2. The effect size results further indicate that digital financial literacy made the largest contribution to trust formation (f^2^ = 0.379), whereas perceived AI advisory transparency showed a smaller but meaningful contribution (f^2^ = 0.096). Together, these two predictors explained 47.1% of the variance in trust in hybrid AI advisory (R^2^ = 0.471), indicating meaningful explanatory power.

For digital wealth management behavior, trust in hybrid AI advisory had a significant positive effect (β = 0.252, t = 3.970, p < 0.001, 95% CI [0.132, 0.381]), supporting H3. Investment and wealth knowledge also positively influenced digital wealth management behavior (β = 0.348, t = 6.092, p < 0.001, 95% CI [0.236, 0.457]), supporting H4. Among the hypothesized predictors of digital wealth management behavior, investment and wealth knowledge showed the stronger direct effect, suggesting that knowledge of financial products, investment strategies, and risk–return trade-offs plays an important role in encouraging active digital wealth management practices. In addition, the interaction term between cyber-risk self-protection and trust in hybrid AI advisory was positive and statistically significant (β = 0.098, t = 2.070, p = 0.038, 95% CI [0.001, 0.183]), supporting H10. However, the effect size of the interaction was small (f^2^ = 0.022), suggesting that cyber-risk self-protection should be interpreted as a modest boundary condition rather than as a central driver of digital wealth management behavior. The predictors jointly explained 60.2% of the variance in digital wealth management behavior (R^2^ = 0.602).

For perceived private wealth management capability, trust in hybrid AI advisory had a significant positive direct effect (β = 0.232, t = 2.699, p = 0.007, 95% CI [0.059, 0.395]), supporting H5. Digital wealth management behavior also positively influenced perceived private wealth management capability (β = 0.332, t = 3.899, p < 0.001, 95% CI [0.162, 0.496]), supporting H6. Investment and wealth knowledge had a significant positive direct effect on perceived private wealth management capability as well (β = 0.205, t = 2.756, p = 0.006, 95% CI [0.055, 0.349]), supporting H7. Although the effect sizes of these predictors were small, the results suggest that perceived private wealth management capability is shaped by complementary mechanisms involving trust, enacted digital behavior, and investment-related knowledge. Collectively, these variables explained 44.3% of the variance in perceived private wealth management capability (R^2^ = 0.443).

The mediation results show that digital wealth management behavior significantly mediated the effect of trust in hybrid AI advisory on perceived private wealth management capability (β_indirect = 0.084, t = 2.641, p = 0.008, 95% CI [0.036, 0.165]), supporting H8. Digital wealth management behavior also mediated the relationship between investment and wealth knowledge and perceived private wealth management capability (β_indirect = 0.116, t = 3.719, p < 0.001, 95% CI [0.062, 0.186]), supporting H9. Because the corresponding direct effects remained positive and statistically significant, both mediating relationships can be interpreted as complementary partial mediation.

The non-hypothesized path from cyber-risk self-protection to digital wealth management behavior was estimated as a lower-order constituent effect required for the moderation model. This path was positive and significant (β = 0.323, t = 5.100, p < 0.001, 95% CI [0.190, 0.439]). However, it was reported for statistical completeness and was not treated as a separate hypothesis. The core substantive interpretation of the model remains centered on the direct and mediated trust-to-capability mechanisms. At the same time, the interaction effect is treated only as a supplementary, modest boundary-condition test.

Taken together, the structural model results support the proposed theoretical framework. Digital financial literacy emerged as the strongest antecedent of trust in hybrid AI advisory, while investment and wealth knowledge were the strongest hypothesized predictors of digital wealth management behavior. Digital wealth management behavior served as an important mechanism through which trust and knowledge were translated into perceived private wealth management capability. Finally, cyber-risk self-protection functioned as a modest but significant boundary condition, indicating that trust in hybrid AI advisory is more likely to translate into active digital wealth management behavior when respondents possess stronger cyber-risk self-protection.

### 4.4 Predictive assessment using PLSpredict

Tables 14 and 15 report the construct- and indicator-level Q^2^predict results. The Q^2^predict statistics for each endogenous construct and its indicators were examined to determine the model’s predictive relevance [32,36]. A Q^2^predict value greater than zero indicates that the model has predictive relevance compared with a naïve benchmark. However, Q^2^predict alone is not sufficient to establish strong out-of-sample predictive performance. Therefore, following the PLSpredict guidelines, the prediction errors of the PLS-SEM model were further compared with those of the linear model (LM) benchmark using indicator-level RMSE values [36].

**Table 14:** Q^2^predict values of the endogenous constructs.

	Q^2^predict
**THA**	0.457
**DWMB**	0.588
**PWMC**	0.439

Note: THA: Trust in Hybrid AI Advisory, DWMB: Digital Wealth Management Behavior, PWMC: Perceived Private Wealth Management Capability, Q^2^predict: predictive relevance.

***Source:*** *Authors’ calculations using SmartPLS 4.1.*

**Table 15:** Indicator-Level Q^2^predict Values.

	Q^2^predict
**THA1**	0.387
**THA2**	0.350
**THA3**	0.286
**DWMB1**	0.541
**DWMB2**	0.478
**DWMB3**	0.494
**PWMC1**	0.376
**PWMC2**	0.318
**PWMC3**	0.356

Note: THA: Trust in Hybrid AI Advisory, DWMB: Digital Wealth Management Behavior, PWMC: Perceived Private Wealth Management Capability, Q^2^predict: predictive relevance.

***Source:*** *Authors’ calculations using SmartPLS 4.1.*

As shown in Table 16, all Q^2^predict values for the key endogenous indicators are positive, ranging from 0.478 to 0.541 for digital wealth management behavior and from 0.318 to 0.376 for perceived private wealth management capability. These results indicate predictive relevance for both the behavioral mechanism and the perceived capability outcome. In addition, the PLS-SEM RMSE values are lower than the corresponding LM RMSE values for all six indicators. This indicates that the PLS-SEM model outperforms the linear benchmark in predicting the key endogenous indicators. The predictive advantage is stronger for digital wealth management behavior, whereas the improvement over the LM benchmark for perceived private wealth management capability is positive but more modest. Therefore, the model demonstrates acceptable out-of-sample predictive performance, with stronger predictive power for digital wealth management behavior than for perceived private wealth management capability.

**Table 16:** Indicator-level plspredict results with linear model benchmark comparison.

Indicator	Q^2^predict	PLS RMSE	LM RMSE	PLS < LM?
DWMB1	0.541	0.920	0.941	Yes
DWMB2	0.478	0.948	0.977	Yes
DWMB3	0.494	0.951	0.963	Yes
PWMC1	0.376	0.873	0.877	Yes
PWMC2	0.318	0.894	0.932	Yes
PWMC3	0.356	0.876	0.883	Yes

Note: DWMB = Digital Wealth Management Behavior; PWMC = Perceived Private Wealth Management Capability; Q^2^predict = predictive relevance; RMSE = root mean square error; PLS = partial least squares structural equation modeling; LM = linear model benchmark.

***Source:*** *Authors’ calculations using SmartPLS 4.1.*

The standardized root mean square residual (SRMR) was examined as a supplementary diagnostic of approximate model fit for the PLS-SEM model. SRMR reflects the average discrepancy between the observed correlation matrix and the model-implied correlation matrix, and values below 0.08 are commonly considered acceptable in empirical SEM applications [40,41]. However, SRMR should be interpreted cautiously in PLS-SEM because PLS-SEM is primarily variance-based and prediction-oriented; therefore, SRMR should not be treated as a definitive global goodness-of-fit criterion in the same way as in covariance-based SEM [31,32,42].

As shown in Table 17, the SRMR values for the saturated model and estimated model were 0.043 and 0.065, respectively. Both values are below the commonly referenced threshold of 0.08. However, the original SRMR values exceeded their corresponding bootstrap-based HI95 and HI99 values. Therefore, the SRMR evidence should be interpreted cautiously. Rather than being used as conclusive evidence of overall model fit, the SRMR result is reported only as a supplementary diagnostic alongside the measurement model results, structural path estimates, explanatory power, effect sizes, mediation and moderation tests, and PLSpredict assessment.

**Table 17:** Supplementary SRMR assessment.

	Original sample (O)	Sample mean (M)	HI95	HI99
**Saturated model**	0.043	0.033	0.038	0.040
**Estimated model**	0.065	0.041	0.049	0.053

Note: SRMR = standardized root mean square residual. HI95 and HI99 refer to the bootstrap-based 95% and 99% quantile values reported by SmartPLS. SRMR is reported as a supplementary approximate fit diagnostic and is not used as a decisive global goodness-of-fit criterion in this PLS-SEM study.

***Source:*** *Authors’ calculations using SmartPLS 4.1.*

### 4.5 Discussion

This study provides a theory-driven explanation of how digitally engaged retail investors develop perceived private wealth management capability in an AI-supported advisory environment. The findings show that a single determinant does not shape perceived private wealth management capability, but rather that it is the combined roles of digital financial literacy, perceived AI advisory transparency, trust in hybrid AI advisory, investment and wealth knowledge, digital wealth management behavior, and cyber-risk self-protection. This result is consistent with the financial capability perspective, which emphasizes that financial capability depends not only on knowledge, but also on the ability to apply knowledge through financial behavior [6,22]. The finding also extends trust in automation theory by showing that trust in hybrid AI advisory becomes meaningful when it is translated into enacted digital wealth management behavior rather than remaining only a favorable attitude toward technology [10,11].

The first major finding concerns the formation of trust in hybrid AI advisory. Digital financial literacy has the strongest effect on trust, suggesting that user competence is a central foundation of informed reliance on AI-supported financial advice. This finding is consistent with the argument that digital financial literacy extends beyond conventional financial literacy, encompassing the ability to evaluate digital financial products, interpret online financial information, and understand the risks associated with digital financial services [7,17]. In the context of hybrid AI advisory, digitally literate investors may be better able to evaluate whether AI-supported recommendations are understandable, credible, and aligned with their financial objectives. Therefore, trust does not appear to emerge from passive acceptance of AI systems, but from users’ ability to interpret and evaluate advisory outputs. This supports the notion of calibrated trust, where users neither reject automated advice automatically nor rely on it unthinkingly [10].

The findings also reinforce recent research emphasizing that AI-supported financial advisory requires a balance between technological capability and human-centered trust calibration. Rather than implying that AI replaces professional advice, the results suggest that hybrid configurations may support investor capability when transparency, knowledge, and behavioral engagement are jointly developed.

Perceived AI advisory transparency also positively influences trust in hybrid AI advisory, although its effect is weaker than that of digital financial literacy. This finding is theoretically meaningful because it suggests that transparency alone may not be sufficient to generate informed trust. Prior research shows that transparency, explainability, and causability can enhance trust and acceptance by helping users understand how AI systems produce recommendations [12,18]. However, the present finding indicates that transparency becomes more valuable when users possess the digital and financial competence needed to interpret explanations. In hybrid AI advisory, transparency should therefore be understood not merely as disclosure, but as a decision-support mechanism that enables investors to connect advisory recommendations with financial goals, risk preferences, and portfolio decisions.

The Vietnamese findings can also be situated against evidence from other emerging-market settings. Research in Malaysia has linked advisory transparency to robo-advisor adoption among B40 users [3]. At the same time, studies in India highlight both the potential of robo-advisory to mitigate investor biases and the continuing value of human involvement in complex financial decisions [20,21]. The present results are consistent with these studies in showing that transparency and trust matter, but extend them by identifying digital wealth management behavior as the mechanism by which trust and investment knowledge translate into perceived capability.

The second major finding concerns digital wealth management behavior. Trust in hybrid AI advisory positively affects digital wealth management behavior, consistent with trust-in-automation theory and prior digital service research, suggesting that trust increases users’ willingness to rely on technology-enabled recommendations [10,26]. However, knowledge of investments and wealth has a stronger effect on digital wealth management behavior than trust does. This distinction is important because it suggests that trust may encourage engagement, but knowledge provides the cognitive foundation for action. Digital wealth management behavior requires investors to monitor portfolios, evaluate risk-return trade-offs, consider advisory inputs, and adjust financial decisions through digital tools. These actions cannot be explained by trust alone.

The third major finding concerns the mediating role of digital wealth management behavior. The results show that digital wealth management behavior partially mediates the effects of both trust in hybrid AI advisory and investment and wealth knowledge on perceived private wealth management capability. This finding supports the financial capability logic that knowledge becomes meaningful when it is translated into behavior [6,22]. It also extends this logic to the AI-supported wealth management context, showing that trust must be enacted through behavior. In other words, investors do not develop stronger perceived private wealth management capability simply because they trust hybrid AI advisory systems. They develop such perceived capability when trust encourages concrete financial actions, including monitoring, use of digital tools, and advisory-based adjustments.

The fourth, more limited finding concerns the modest boundary-conditioning role of cyber-risk self-protection. The interaction between cyber-risk self-protection and trust in hybrid AI advisory is positive and statistically significant, but the effect size is small. This finding is consistent with protection motivation theory, which emphasizes that individuals are more likely to take protective or technology-related actions when they believe they can cope with potential threats [13,14]. It is also consistent with recent information security research showing that coping appraisal and self-efficacy are important drivers of security-related behavior [16]. In the present study, cyber-risk self-protection provides supportive but limited evidence that protective digital capabilities may help investors translate trust into digital wealth-management behavior. Therefore, this result should be interpreted as a secondary boundary condition, not as the main mechanism explaining digital wealth management behavior.

Taken together, the findings advance the literature by reframing hybrid AI advisory as a trust-to-capability process. Prior robo-advisory research has mainly focused on adoption, automation, and platform use [19–21]. The present study extends this stream by showing that the value of hybrid AI advisory lies not only in whether investors accept or trust the advisory system, but also in whether that trust translates into responsible digital wealth management behavior. This distinction is particularly relevant in emerging markets, where digital financial services are expanding rapidly but investors may differ substantially in digital literacy, investment knowledge, and cyber-risk self-protection.

## 5 Theoretical contributions

This study makes three theoretical contributions by extending the Financial Capability Framework, Trust in Automation Theory, and Protection Motivation Theory into the context of hybrid artificial intelligence advisory and digital wealth management in an emerging market.

### 5.1 Contribution to the financial capability framework

First, this study extends the Financial Capability Framework by showing that perceived private wealth management capability is shaped not solely by investment knowledge but by the interaction between knowledge-based resources and enacted digital wealth management behavior. Prior research on financial capability emphasizes that financial knowledge becomes meaningful when it translates into financial behavior [6,22]. The present study advances this logic by situating the knowledge-behavior-capability process within an AI-supported digital advisory environment. The findings show that investment and wealth knowledge positively affect digital wealth management behavior and perceived private wealth management capability. At the same time, digital wealth management behavior also mediates the relationship between investment and wealth knowledge and perceived capability. This indicates that capability development in digital wealth management requires both cognitive understanding and behavioral enactment.

This contribution is important because digital wealth management differs from conventional personal finance settings. Investors are no longer required to understand only financial products or investment principles; they must also monitor digital portfolios, interpret advisory recommendations, and use technology-enabled tools to act on financial information. By demonstrating that digital wealth management behavior functions as a behavioral mechanism linking knowledge to perceived capability, the study refines the Financial Capability Framework for digital and AI-assisted financial environments. It suggests that financial capability should be understood as a dynamic process in which knowledge is activated through repeated digital financial practices rather than as a static stock of financial literacy.

### 5.2 Contribution to trust in automation theory

Second, this study extends Trust in Automation Theory by shifting the focus from trust formation and technology acceptance to the conversion of calibrated trust into responsible digital wealth management behavior. Trust in Automation Theory argues that trust is most important when users face uncertainty, vulnerability, and limited ability to verify system processes directly [10]. In hybrid AI advisory, this condition is particularly relevant because users must evaluate recommendations produced through the combined roles of algorithmic analysis and human advisory support. The findings show that digital financial literacy and perceived AI advisory transparency significantly shape trust in hybrid AI advisory, indicating that trust is influenced by both user-side competence and system-side transparency.

More importantly, the study contributes to Trust in Automation Theory by showing that trust is not only an attitudinal outcome but also a behavioral antecedent. Trust in hybrid AI advisory positively affects digital wealth management behavior and perceived private wealth management capability, and its effect on perceived capability is partially mediated by digital wealth management behavior. This finding supports the idea of calibrated reliance: trust is theoretically valuable when it leads users to engage in appropriate financial actions rather than passively accepting or unthinkingly complying. Therefore, the study moves beyond adoption-oriented explanations of robo-advisory use and demonstrates that the central theoretical issue in hybrid AI advisory is not simply whether investors trust the system, but whether that trust translates into disciplined portfolio monitoring, advisory-based adjustments, and responsible use of digital financial tools.

### 5.3 Contribution to protection motivation theory

Third, this study extends Protection Motivation Theory by providing modest boundary-condition evidence for the role of cyber-risk self-protection in the trust-behavior relationship. Protection Motivation Theory explains protective behavior through threat appraisal and coping appraisal, particularly individuals’ perceived ability to perform effective protective actions [13,14]. Information security research has similarly shown that coping appraisal variables, including response efficacy and self-efficacy, are important predictors of security-related behavior [16]. The present study extends this reasoning by arguing that cyber-risk self-protection is not only a direct security-related capability but also a supplementary condition that may affect whether trust in hybrid AI advisory becomes actionable.

The findings indicate that cyber-risk self-protection positively moderates the relationship between trust in hybrid AI advisory and digital wealth management behavior. However, because the moderating effect is small, this contribution should be interpreted with caution. Rather than positioning cyber-risk self-protection as the model’s central mechanism, the study suggests that protective capability serves as a secondary enabling condition, making trust more actionable in digital wealth management contexts. In digital wealth management, protective capability may reduce hesitation associated with cyber-risk exposure. Still, the principal explanatory pathway remains the conversion of trust and knowledge into enacted digital wealth management behavior.

These three contributions advance the literature by reframing hybrid AI advisory as a trust-to-capability process for digitally engaged retail investors. The study integrates financial capability, trust calibration, behavioral enactment, and cyber-risk protection into a single explanatory model. This theoretical integration suggests that perceived private wealth management capability in AI-assisted retail-investor environments depends not only on what investors know or whether they trust the system, but also on whether trust and knowledge are converted into consistent digital wealth management behavior under conditions of perceived cyber-risk self-protection.

## 6 Managerial implications and recommendations

The findings offer practical implications for financial institutions, fintech providers, and digital wealth advisory platforms serving retail investors. Hybrid AI advisory systems should be designed not only to be transparent and trustworthy but also to support users in understanding recommendations, monitor portfolios, and manage digital financial risks. Investor education programs should also integrate digital financial literacy, investment knowledge, and cyber-risk self-protection rather than treating them as separate capabilities.

### 6.1 Developing digital financial literacy as trust infrastructure

Digital financial literacy has the strongest effect on trust in hybrid artificial intelligence advisory (β = 0.517, f^2^ = 0.379). This result suggests that trust should not be treated solely as a platform-design issue. It is also shaped by users’ ability to assess digital financial information, understand technology-enabled recommendations, and recognize the opportunities and limitations of digital financial services.

Financial institutions should therefore embed digital financial literacy development into the user journey. At the onboarding stage, users could complete a brief capability assessment covering digital platform use, interpretation of investment information, recognition of risk, and familiarity with advisory tools. The results could be used to provide personalized learning modules, short investment simulations, and contextual explanations before users interact with advanced advisory functions.

This recommendation is consistent with the broader view that digital financial literacy extends beyond conventional financial knowledge and includes the competencies required to navigate digital financial environments safely and effectively [7,8]. Evidence that digital financial literacy is associated with saving and spending behavior further supports the value of practical, behavior-oriented learning interventions rather than passive educational materials alone [9]. For providers, digital financial education should therefore be treated as part of service quality and trust formation rather than as an optional corporate social responsibility activity.

### 6.2 Designing transparent and calibrated hybrid artificial intelligence advisory

Perceived artificial intelligence advisory transparency positively affects trust in hybrid artificial intelligence advisory (β = 0.261, f^2^ = 0.096). Although its effect is smaller than that of digital financial literacy, transparency remains an important system-level mechanism for reducing uncertainty and facilitating informed reliance.

Hybrid advisory platforms should provide understandable, decision-relevant explanations that are proportionate to the complexity of the recommendation. Users should be able to identify why a particular portfolio adjustment is suggested, which assumptions are used, how risk is assessed, and under what conditions the recommendation may no longer be appropriate. Scenario analysis, plain-language summaries, visual explanations of risk–return trade-offs, and disclosure of uncertainty ranges can make advisory outputs more interpretable.

Transparency should not be equated with the disclosure of technically complex algorithms. Excessive technical detail may increase cognitive burden without improving decision quality. The managerial objective is calibrated transparency: enough information to support informed judgment, but presented in a form that users can use effectively. Prior research indicates that explainability and causability influence users’ perceptions, trust, and acceptance of artificial intelligence systems [12]. The broader trust-in-artificial-intelligence literature also shows that trust is shaped by multiple technological and contextual cues rather than by system performance alone [11].

Hybrid platforms should additionally retain clear opportunities for human escalation. Users should be able to consult a human advisor when recommendations involve major portfolio adjustments, unusual market conditions, or financial decisions with long-term consequences. Human oversight can reinforce appropriate reliance without undermining the efficiency advantages of artificial intelligence.

### 6.3 Converting knowledge and trust into sustained digital wealth management behavior

Investment and wealth knowledge positively influence digital wealth management behavior (β = 0.348, f^2^ = 0.189), while trust in hybrid artificial intelligence advisory also contributes to such behavior (β = 0.252, f^2^ = 0.092). More importantly, digital wealth management behavior partially mediates the relationships between trust and perceived private wealth management capability, as well as between investment and wealth knowledge and perceived private wealth management capability.

These findings indicate that knowledge and trust are necessary but insufficient. Their practical value emerges when retail investors convert them into repeated actions, such as monitoring portfolios, reviewing risk exposure, adjusting asset allocations, and responding appropriately to advisory recommendations. Digital wealth advisory platforms should therefore design services that support behavioral enactment rather than merely provide information.

Useful features include personalized portfolio reviews, timely monitoring alerts, scenario-based prompts, progress dashboards, and reminders to reassess financial goals. Such tools should be configured carefully to prevent overtrading or excessive dependence on automated recommendations. The objective is to encourage disciplined and goal-consistent behavior, not frequent activity for its own sake.

Providers should also distinguish between informative nudges and prescriptive automation. For lower-risk actions, such as reviewing portfolio allocations or updating financial goals, prompts may be sufficient. For higher-stakes decisions, the system should provide explanatory content and opportunities for human consultation. This staged approach recognizes that capability develops when users understand recommendations, reflect on their implications, and act consistently over time.

### 6.4 Embedding cyber-risk self-protection into the advisory journey

Cyber-risk self-protection positively moderates the relationship between trust in hybrid artificial intelligence advisory and digital wealth management behavior (β = 0.098, f^2^ = 0.022). The moderation effect is statistically significant but modest in magnitude. It should therefore be interpreted as a modest boundary condition that supports the trust-to-behavior pathway, rather than as a dominant mechanism in the model.

The result suggests that retail investors may trust a hybrid advisory system but still hesitate to act if they feel unable to protect their accounts, identify suspicious activities, or manage digital threats. Financial institutions should embed cybersecurity support directly into the advisory experience. Security communication should be timely, contextual, and linked to the actions users are expected to perform.

Practical interventions include short phishing-recognition exercises, contextual warnings before sensitive transactions, multi-factor authentication prompts, device-management dashboards, and clear guidance for responding to suspicious activities. Security education should avoid fear-based messaging that overwhelms users or discourages digital participation. Instead, providers should emphasize actionable coping strategies and users’ ability to perform them.

This recommendation is consistent with research on Protection Motivation Theory in information security. A meta-analytic assessment by [16] shows that coping appraisal variables, including response efficacy and self-efficacy, play important roles in security-related behavior. On wealth management platforms, cybersecurity should therefore be treated not only as a technical infrastructure issue but also as a retail investor capability issue.

### 6.5 Implementing an integrated and segmented retail-investor support strategy

Perceived private wealth management capability is shaped jointly by trust in hybrid artificial-intelligence advisory services, digital wealth management behavior, and investment and wealth knowledge. The findings therefore support an integrated retail-investor support strategy rather than isolated interventions. Financial institutions should assess users’ capabilities, provide appropriate educational support, explain recommendations transparently, facilitate disciplined action, and reinforce safe digital engagement throughout the service journey.

A segmented approach can improve the efficiency of this strategy. Users with limited digital financial literacy may benefit from guided onboarding, simplified explanations, and gradual exposure to advanced advisory features. Users with stronger investment knowledge but lower behavioral engagement may require personalized monitoring tools and structured portfolio-review prompts. Users who demonstrate high trust but weaker cyber-risk self-protection may need targeted security support before being encouraged to execute digital transactions independently.

The segmentation logic should remain dynamic. Providers can use users’ interaction patterns, completion of learning modules, portfolio-review behavior, and security practices to refine the level of support offered over time. However, personalization should remain transparent and proportionate, with appropriate safeguards for privacy and human oversight.

Taken together, the findings suggest that the effectiveness of hybrid artificial intelligence advisory does not depend solely on technological sophistication. Sustainable retail-investor capability outcomes are more likely to emerge when providers combine digital financial education, explainable advisory processes, behavior-support tools, and embedded cybersecurity guidance. This integrated approach can help retail investors develop informed trust, translate advisory insights into consistent wealth-management behavior, and strengthen their long-term perceived capability in private wealth management.

### 6.6 Policy implications

At the policy level, financial-market and consumer-protection authorities could encourage proportionate disclosure standards for AI-supported financial advice, support digital financial literacy and cybersecurity education, and preserve access to human review for higher-stakes advisory decisions. These implications follow from the evidence that responsible engagement depends on both system transparency and users’ capability to interpret, act on, and safely implement advisory recommendations; they should therefore be treated as enabling safeguards rather than as claims about objective investment performance.

## 7 Conclusion

This study examined how digital financial literacy, perceived AI advisory transparency, investment and wealth knowledge, and cyber-risk self-protection shape perceived private wealth management capability among digitally engaged retail investors in an emerging-market context. By integrating the Financial Capability Framework, Trust in Automation Theory, and Protection Motivation Theory, the study proposed and tested a trust-to-capability model in which trust in hybrid AI advisory and digital wealth management behavior serves as a key explanatory mechanism.

The findings show that digital financial literacy is the strongest antecedent of trust in hybrid AI advisory, while perceived AI advisory transparency also plays a significant role in shaping trust. Investment and wealth knowledge emerges as the strongest hypothesized predictor of digital wealth management behavior. Trust, digital wealth management behavior, and investment and wealth knowledge all positively affect perceived private wealth management capability. In addition, digital wealth management behavior partially mediates the effects of both trust and investment and wealth knowledge on perceived capability. Cyber-risk self-protection provides modest support as a boundary condition in the trust-behavior relationship, and its small moderating effect should be interpreted cautiously.

These results suggest that perceived private wealth management capability in AI-supported financial environments is not determined solely by financial knowledge, trust, or technology use. Rather, capability develops when investors combine digital financial literacy, informed trust, investment knowledge, protective cyber-risk awareness, and consistent digital wealth management behavior. The study, therefore, highlights the importance of moving beyond simple robo-advisory adoption and examining how trust in hybrid AI advisory is translated into responsible financial action.

The findings offer practical implications for financial institutions, fintech providers, and advisory platforms. Hybrid AI advisory systems should be designed not only to be transparent and trustworthy, but also to help users understand recommendations, monitor portfolios, and manage digital financial risks. Investor education programs should also integrate digital financial literacy, investment knowledge, and cyber-risk self-protection rather than treating them as separate capabilities.

## 8 Limitations and future research directions

This study has several limitations. The data were collected from a non-probability sample of digitally engaged retail investors in Vietnam, and the findings should not be generalized to all investors, private banking clients, or high-net-worth individuals. The study also relied on cross-sectional self-reported data; therefore, perceived private wealth management capability should be interpreted as self-assessed capability rather than as objective portfolio performance, verified investment skill, private banking eligibility, or realized wealth outcomes. Future research could use longitudinal designs, objective investment-performance indicators, cross-country samples, or experimental approaches to examine how hybrid AI advisory, calibrated trust, and cyber-risk self-protection influence actual wealth management decisions over time.

Additionally, the relatively small sample size and digitally engaged respondent profile may introduce selection bias, as individuals with a greater interest in digital financial services may be overrepresented. Although common method bias was assessed using multiple statistical procedures, it cannot be fully ruled out given the cross-sectional and self-reported nature of the study design.

## Supporting information

S1 File Anonymized dataset. Dataset underlying the analyses reported in the manuscript.
(XLSX)

S2 File Codebook. Variable names, coding, and construct/item labels corresponding to the anonymized dataset.
(XLSX)

S3 File Survey questionnaire. Questionnaire used for participant screening, demographic information, and measurement of the seven study constructs.
(PDF)

S4 File Analysis outputs. Supplementary outputs supporting the PLS-SEM analyses reported in the manuscript.
(7Z)
