# Supplementary material for: Digital financial literacy and trust in hybrid AI advisory: Explaining perceived private wealth management capability among digitally engaged retail investors in an emerging market
Source: PLoS One. 2026 Oct 30;21(10):e0353922. doi: 10.1371/journal.pone.0353922 (PMC13722208; doi:10.1371/journal.pone.0353922)
Supplement: Supplementary file 1 [file pone.0353922.s003.pdf]

SURVEY QUESTIONNAIRE

(BẢNG CÂU HỎI KHẢO SÁT)

Digital Financial Literacy and Trust in Hybrid AI Advisory: Explaining Perceived Private Wealth Management Capability Among Digitally Engaged Retail Investors in an Emerging Market

(Hiểu biết tài chính số và niềm tin vào tư vấn AI kết hợp: Giải thích năng lực quản lý tài sản cá nhân được cảm nhận của các nhà đầu tư cá nhân tích cực sử dụng công nghệ số tại một thị trường mới nổi)

Dear Participant,

(Kính gửi Quý Anh/Chị,)

We are conducting an academic study examining how digital financial literacy, perceived AI advisory transparency, investment and wealth knowledge, cyber-risk self-protection, trust in hybrid AI advisory, and digital wealth management behavior relate to perceived private wealth management capability among digitally engaged retail investors in Vietnam.

(Chúng tôi đang thực hiện một nghiên cứu học thuật nhằm xem xét mối quan hệ giữa hiểu biết tài chính số, nhận thức về tính minh bạch của tư vấn AI, kiến thức đầu tư và quản lý tài sản, khả năng tự bảo vệ trước rủi ro an ninh mạng, niềm tin vào tư vấn AI kết hợp, hành vi quản lý tài sản số và năng lực quản lý tài sản cá nhân được cảm nhận của các nhà đầu tư cá nhân tích cực sử dụng công nghệ số tại Việt Nam.)

We kindly invite you to take a few minutes to complete this questionnaire. Your responses will be used solely for academic research. Participation is voluntary. No names, email addresses, telephone numbers, IP addresses, or other personally identifiable information are collected. You may discontinue the survey at any time before submitting your responses.

(Kính mong Quý Anh/Chị dành vài phút để hoàn thành bảng câu hỏi này. Các câu trả lời chỉ được sử dụng cho mục đích nghiên cứu học thuật. Việc tham gia hoàn toàn tự nguyện. Nghiên cứu không thu thập họ tên, địa chỉ email, số điện thoại, địa chỉ IP hoặc bất kỳ thông tin nhận dạng cá nhân nào khác. Quý Anh/Chị có thể dừng tham gia khảo sát bất kỳ lúc nào trước khi gửi câu trả lời.)

By selecting "Continue" after the consent statement, you confirm that you are at least 18 years old, have read the information above, and voluntarily agree to participate in the study.

(Bằng cách chọn "Tiếp tục" sau phần xác nhận đồng thuận, Quý Anh/Chị xác nhận rằng mình từ 18 tuổi trở lên, đã đọc các thông tin nêu trên và tự nguyện đồng ý tham gia nghiên cứu.)

Thank you very much for your time and valuable contribution to this research.

(Xin chân thành cảm ơn Quý Anh/Chị đã dành thời gian và đóng góp ý kiến quý báu cho nghiên cứu này.)

Sincerely,

(Trân trọng,)

The Research Team

(Nhóm nghiên cứu)

I. ELIGIBILITY SCREENING

(I. CÂU HỎI SÀNG LỌC)

Please select one answer for each statement below.

(Vui lòng chọn một phương án trả lời cho mỗi câu hỏi dưới đây.)

**Code** *(Mã)*	**Screening question** *(Câu hỏi sàng lọc)*	**Yes** *(Có)*	**No** *(Không)*
S1	Are you 18 years of age or older? *(Anh/Chị có từ 18 tuổi trở lên không?)*	☐ Yes *(☐ Có)*	☐ No *(☐ Không)*
S2	Do you currently have an active bank, securities, mutual fund, or digital investment account? *(Hiện tại Anh/Chị có tài khoản ngân hàng, tài khoản chứng khoán, quỹ tương hỗ hoặc tài khoản đầu tư số đang hoạt động không?)*	☐ Yes *(☐ Có)*	☐ No *(☐ Không)*
S3	Have you previously used a digital financial platform, investment application, robo-advisory tool, or AI-supported advisory function? *(Anh/Chị đã từng sử dụng nền tảng tài chính số, ứng dụng đầu tư, công cụ tư vấn tự động (robo-advisory) hoặc chức năng tư vấn có hỗ trợ AI chưa?)*	☐ Yes *(☐ Có)*	☐ No *(☐ Không)*
S4	Do you have experience monitoring, allocating, or adjusting personal financial assets? *(Anh/Chị có kinh nghiệm theo dõi, phân bổ hoặc điều chỉnh tài sản tài chính cá nhân không?)*	☐ Yes *(☐ Có)*	☐ No *(☐ Không)*
S5	Do you have investable financial assets or recurring savings available for investment? *(Anh/Chị có tài sản tài chính có thể đầu tư hoặc khoản tiết kiệm định kỳ dành cho đầu tư không?)*	☐ Yes *(☐ Có)*	☐ No *(☐ Không)*

Eligibility notes: Only respondents who answered “Yes” to all screening questions were eligible to proceed to the main questionnaire.

(Lưu ý về điều kiện tham gia: Chỉ những người trả lời “Có” cho tất cả các câu hỏi sàng lọc mới đủ điều kiện tiếp tục thực hiện bảng câu hỏi chính.)

II. GENERAL INFORMATION

(II. THÔNG TIN CHUNG)

Please tick the option that best describes you.

(Vui lòng đánh dấu vào phương án phù hợp nhất với Quý Anh/Chị.)

1. Gender

(1. Giới tính)

☐ Male ☐ Female

(☐ Nam ☐ Nữ)

2. Age

(2. Độ tuổi)

☐ 18–29 ☐ 30–39 ☐ 40–45 ☐ Over 45

(☐ 18–29 ☐ 30–39 ☐ 40–45 ☐ Trên 45)

3. Highest education level

(3. Trình độ học vấn cao nhất)

☐ Bachelor ☐ Postgraduate ☐ College/Intermediate ☐ High School ☐ Other

(☐ Đại học ☐ Sau đại học ☐ Cao đẳng/Trung cấp ☐ Trung học phổ thông ☐ Khác)

4. Average monthly income

(4. Thu nhập trung bình hàng tháng)

☐ < $200 ☐ $200–$400 ☐ $400–$800 ☐ $800–$1,000 ☐ > $1,000

(☐ < 200 USD ☐ 200–400 USD ☐ 400–800 USD ☐ 800–1.000 USD ☐ > 1.000 USD)

5. Investment experience

(5. Kinh nghiệm đầu tư)

☐ < 1 year ☐ 1–3 years ☐ 3–5 years ☐ 5–7 years ☐ > 7 years

(☐ < 1 năm ☐ 1–3 năm ☐ 3–5 năm ☐ 5–7 năm ☐ > 7 năm)

6. Investment risk tolerance

(6. Mức chấp nhận rủi ro đầu tư)

☐ Very Low ☐ Low ☐ Medium ☐ High ☐ Very High

(☐ Rất thấp ☐ Thấp ☐ Trung bình ☐ Cao ☐ Rất cao)

III. SURVEY CONTENT

(III. NỘI DUNG KHẢO SÁT)

Please indicate your level of agreement with each statement by selecting the number that best represents your opinion:

(Vui lòng cho biết mức độ đồng ý của Quý Anh/Chị đối với từng phát biểu bằng cách chọn con số phù hợp nhất với ý kiến của mình:)

1 – Strongly disagree

(1 – Hoàn toàn không đồng ý)

2 – Disagree

(2 – Không đồng ý)

3 – Neutral

(3 – Trung lập)

4 – Agree

(4 – Đồng ý)

5 – Strongly agree

(5 – Hoàn toàn đồng ý)

**Code** *(Mã)*	**Statement** *(Phát biểu)*	**1**	**2**	**3**	**4**	**5**
**I. Digital Financial Literacy (DFL)** ***(I. Hiểu biết tài chính số (DFL))***						
DFL1	I can evaluate digital financial products effectively. *(Tôi có thể đánh giá các sản phẩm tài chính số một cách hiệu quả.)*	1	2	3	4	5
DFL2	I understand how to use online investment platforms. *(Tôi hiểu cách sử dụng các nền tảng đầu tư trực tuyến.)*	1	2	3	4	5
DFL3	I can critically assess digital financial information. *(Tôi có thể đánh giá một cách phản biện các thông tin tài chính số.)*	1	2	3	4	5
**II. Perceived AI Advisory Transparency (PAAT)** ***(II. Nhận thức về tính minh bạch của tư vấn AI (PAAT))***						
PAAT1	AI recommendations are clearly explained. *(Các khuyến nghị của AI được giải thích rõ ràng.)*	1	2	3	4	5
PAAT2	I understand how AI generates financial suggestions. *(Tôi hiểu cách AI tạo ra các gợi ý tài chính.)*	1	2	3	4	5
PAAT3	The AI advisory process is transparent and understandable. *(Quy trình tư vấn của AI minh bạch và dễ hiểu.)*	1	2	3	4	5
**III. Trust in Hybrid AI Advisory (THA)** ***(III. Niềm tin vào tư vấn AI kết hợp (THA))***						
THA1	I trust that AI + human advisory provides reliable recommendations.	1	2	3	4	5

	(Tôi tin tưởng rằng tư vấn kết hợp giữa AI và con người đưa ra các khuyến nghị đáng tin cậy.)					
THA2	I believe the hybrid advisory system is competent. *(Tôi tin rằng hệ thống tư vấn kết hợp có năng lực.)*	1	2	3	4	5
THA3	I feel confident relying on the hybrid AI advisory. *(Tôi cảm thấy tin tưởng khi dựa vào tư vấn AI kết hợp.)*	1	2	3	4	5
**IV. Investment and Wealth Knowledge (IWK)** ***(IV. Kiến thức đầu tư và quản lý tài sản (IWK))***						
IWK1	I understand financial products and investment strategies. *(Tôi hiểu các sản phẩm tài chính và chiến lược đầu tư.)*	1	2	3	4	5
IWK2	I can evaluate investment opportunities effectively. *(Tôi có thể đánh giá các cơ hội đầu tư một cách hiệu quả.)*	1	2	3	4	5
IWK3	I am knowledgeable about risk-return trade-offs. *(Tôi có kiến thức về sự đánh đổi giữa rủi ro và lợi nhuận.)*	1	2	3	4	5
**V. Digital Wealth Management Behavior (DWMB)** ***(V. Hành vi quản lý tài sản số (DWMB))***						
DWMB1	I regularly monitor and adjust my digital investment portfolio. *(Tôi thường xuyên theo dõi và điều chỉnh danh mục đầu tư trên nền tảng số của mình.)*	1	2	3	4	5
DWMB2	I consider AI or human advisory recommendations consistently. *(Tôi cân nhắc một cách nhất quán các khuyến nghị tư vấn từ AI hoặc con người.)*	1	2	3	4	5
DWMB3	I use digital tools to manage my wealth proactively. *(Tôi chủ động sử dụng các công cụ số để quản lý tài sản của mình.)*	1	2	3	4	5
**VI. Cyber-risk Self-Protection (CSP)** ***(VI. Khả năng tự bảo vệ trước rủi ro an ninh mạng (CSP))***						
CSP1	I take precautions to secure my online financial accounts. *(Tôi thực hiện các biện pháp phòng ngừa để bảo mật các tài khoản tài chính trực tuyến của mình.)*	1	2	3	4	5
CSP2	I am able to recognize potential online financial threats. *(Tôi có khả năng nhận diện các mối đe dọa tài chính trực*	1	2	3	4	5

	tuyến tiềm ẩn.)					
CSP3	I actively prevent cybersecurity risks when using financial platforms.<br>*(Tôi chủ động phòng ngừa các rủi ro an ninh mạng khi sử dụng các nền tảng tài chính.)*	1	2	3	4	5
**VII. Perceived Private Wealth Management Capability (PWMC)**<br>***(VII. Năng lực quản lý tài sản cá nhân được cảm nhận (PWMC))***						
PWMC 1	I am capable of managing my personal wealth effectively.<br>*(Tôi có khả năng quản lý tài sản cá nhân của mình một cách hiệu quả.)*	1	2	3	4	5
PWMC 2	I can optimize financial outcomes using knowledge and behavior.<br>*(Tôi có thể tối ưu hóa kết quả tài chính bằng cách vận dụng kiến thức và hành vi.)*	1	2	3	4	5
PWMC 3	My wealth management decisions are informed and consistent.<br>*(Các quyết định quản lý tài sản của tôi có đầy đủ căn cứ thông tin và nhất quán.)*	1	2	3	4	5

Thank you sincerely for your participation. Your responses are valuable to this academic research. *(Xin chân thành cảm ơn sự tham gia của Quý Anh/Chị. Những câu trả lời của Quý Anh/Chị có ý nghĩa rất quan trọng đối với nghiên cứu học thuật này.)*
