# Supplementary material for: Digital financial literacy and trust in hybrid AI advisory: Explaining perceived private wealth management capability among digitally engaged retail investors in an emerging market
Source: PLoS One. 2026 Oct 30;21(10):e0353922. doi: 10.1371/journal.pone.0353922 (PMC13722208; doi:10.1371/journal.pone.0353922)
Supplement: Supplementary file 2 [file pone.0353922.s006.pdf]

Response to Academic Editor and Reviewers

Dear Academic Editor and Reviewers,

We sincerely thank the Academic Editor and the three reviewers for their careful and constructive comments. Because the decision is a minor revision, we revised the manuscript conservatively. The conceptual model, hypotheses, sample (N = 249), measurement model, and reported structural results were not altered. The revision is limited to the points raised in the decision letter: clarification of the objective and theoretical positioning, additional methodological transparency, clearer questionnaire-source reporting, explicit internal-consistency wording, targeted comparison with prior and cross-country research, separation of policy implications and future research, table cross-references, and Supporting Information labeling. All additions and modifications are shown in red in the marked-up manuscript, and a clean version is also provided.

A. Academic Editor and Journal Requirements

Requirement 1 – PLOS ONE style and file naming

We sincerely appreciate the Academic Editor's guidance on PLOS ONE style and file-naming requirements. In response, we reviewed the revision package and prepared separate clean and marked-up manuscript files using clear PLOS ONE-oriented filenames. We deliberately avoided unnecessary stylistic rewriting so that the revision remains focused on the issues raised in the decision letter.

Requirement 2 – Financial disclosure and role of funders

We sincerely thank the Academic Editor for requesting clarification of the financial disclosure and the role of the funders. The revised cover letter now states: "This research was partially funded by Ho Chi Minh City University of Economics and Finance (UEF), Ho Chi Minh City, Vietnam, and partially funded by Tomas Bata University in Zlín, Czech Republic, through research project RO/FaME/2026/03. The funders had no role in study design, data collection and analysis, decision to publish, or preparation of the manuscript."

Requirement 3 – In-text references to tables

We sincerely appreciate the Academic Editor for drawing our attention to the table cross-references. We added explicit narrative references to Tables 1, 2, 3, 4, 5, 6, 8, 9, 10, 11, 12, 13, and 14. We also corrected the effect-size paragraph from "Table 7" to "Table 9." These revisions are limited to cross-referencing and do not alter any table values or analytical results.

Requirement 4 – Supporting Information captions

We sincerely thank the Academic Editor for this helpful requirement. We added matching Supporting Information captions for S1–S4 at the end of the manuscript. The Data Availability Statement also states that the anonymized dataset, codebook, survey questionnaire, and analysis outputs are provided as Supporting Information.

Requirement 5 – Suggested citations

We sincerely appreciate the Academic Editor's guidance regarding suggested citations. No reviewer supplied a mandatory citation. We therefore avoided adding publications solely for compliance and retained a focused reference base. To address the request for recent literature and international comparison, we used relevant references already present in the submitted bibliography.

Requirement 6 – Reference-list review

We sincerely thank the Academic Editor for this important reminder. We reviewed the reference list for completeness, consistency, and relevance. The revision did not introduce unnecessary reference renumbering or replace foundational theoretical sources solely because they are older.

Additional Editor Comment – Share the study questionnaire

We sincerely thank the Academic Editor for this valuable suggestion. The study questionnaire is now provided as S3 File in the Supporting Information, with a matching caption at the end of the manuscript. The Data Availability Statement also confirms that the survey questionnaire is included among the Supporting Information materials.

B. Reviewer #1

Comment: Please run Harman's single-factor test or report full-collinearity VIFs, ensuring values below 3.3.

Response: We sincerely thank Reviewer #1 for highlighting the importance of assessing potential common method bias. Both diagnostics had already been conducted and are retained in the revised manuscript. Harman's single-factor test shows that the first factor explains 50.823% of the variance; we report this transparently as a borderline result rather than as evidence that common method bias is absent. The complementary full-collinearity VIF values range from 1.761 to 2.646, all below the conservative 3.3 threshold. Taken together, these results suggest that severe common method bias is unlikely to dominate the structural relationships, while we acknowledge that it cannot be completely ruled out in a cross-sectional self-report design. To make the evidence easier to locate, the revised text now explicitly refers to Tables 5 and 6. No new model estimation was introduced.

Comment – Data Availability

Reviewer #1 indicated that the data underlying the findings had not been fully made available.

Response: We sincerely thank Reviewer #1 for drawing attention to data availability. We have addressed this concern by providing the anonymized respondent-level dataset as S1 File, the corresponding codebook as S2 File, the study questionnaire as S3 File, and the analysis outputs as S4 File. The Data Availability Statement has also been updated to clarify that these materials are provided as Supporting Information.

C. Reviewer #2

Comment 1 – Describe the field-study methodological procedures in greater detail.

Response: We sincerely thank Reviewer #2 for this constructive suggestion. We added a concise paragraph to Section 3.3 describing the actual sequence of the online field procedure: informed consent, eligibility screening, completion of demographic and construct measures by eligible respondents, and completeness screening after data collection. The previously reported dates, recruitment channels, ethics information, exclusion of 19 incomplete responses, and final sample of N = 249 remain unchanged.

Comment 2 – Clearly identify the source of the questionnaire and justify its adoption.

Response: We sincerely thank Reviewer #2 for emphasizing the need to clarify the questionnaire sources and their rationale. We now explain in Section 3.2 that the questionnaire was not adopted wholesale from a single instrument. Instead, construct-specific items were adapted and contextualized from the published sources already cited for each construct. We also added a concise rationale explaining that these sources correspond to the conceptual domains represented in the model and that the wording was contextualized to hybrid AI-supported wealth management while retaining the conceptual meaning of the source constructs.

Comment 3 – Add internal-consistency validity and describe expert-panel review.

Response: We sincerely thank Reviewer #2 for this important comment on instrument development and internal consistency. We agree that these aspects should be reported transparently. However, a formal expert-panel review was not part of the original study procedure; therefore, we have not retrospectively reported an expert-panel validation step that did not occur. Instead, the manuscript reports the procedures actually undertaken: source-based adaptation, item-by-item comparison of the English and Vietnamese versions, and a pilot test with 10 eligible Vietnamese individual investors. We also revised the Section 4.2.1 heading to explicitly include "Internal Consistency." The manuscript reports Cronbach's alpha values of 0.830–0.918, rho_A values of 0.835–0.921, and composite reliability values of 0.898–0.948, supporting satisfactory internal consistency.

Comment 4 – Expand discussion of hypothesis testing and compare with previous studies.

Response: We sincerely thank Reviewer #2 for encouraging a stronger comparison between the hypothesis-testing results and prior research. The Discussion already interprets the principal direct, mediating, and moderating findings against the relevant theoretical literature. To address the comment directly while keeping the minor revision focused, we added a targeted cross-country comparison paragraph that situates the Vietnamese findings alongside Malaysian robo-advisory evidence on transparency and Indian studies on robo-advisory, investor bias, and human involvement, using references [3], [20], and [21] already present in the bibliography.

D. Reviewer #3

Comment 1 – Clarify the study objective.

Response: We sincerely thank Reviewer #3 for pointing out the need for a clearer and more in-depth study objective. We added a dedicated paragraph in the Introduction that states the primary objective and identifies three specific analytical questions: the antecedents of trust, the direct and mediated trust/knowledge-to-capability pathways through digital wealth management behavior, and the moderating role of cyber-risk self-protection.

Comments 2 and 5 – Strengthen theoretical support in the Introduction and Literature Review.

Response: We sincerely thank Reviewer #3 for emphasizing the importance of stronger theoretical support. The Literature Review already contains dedicated sections on the Financial Capability Framework, Trust in Automation Theory, Protection Motivation Theory, and their integration. To make the theoretical logic more explicit earlier in the manuscript, the new objective paragraph in the Introduction now states the distinct role of each theory in the proposed model. We therefore retained the existing theoretical review and strengthened the earlier positioning without introducing unnecessary restructuring.

Comment 3 – Add recent citations from 2020–2025.

Response: We sincerely thank Reviewer #3 for recommending greater visibility of recent literature. The submitted literature review already included multiple sources published between 2020 and 2024. To respond directly to the comment, we added a concise 2025 empirical statement in the literature review using reference [2], which was already included in the bibliography. Foundational older works are retained where necessary to establish the original theoretical constructs.

Comment 4 – Provide a more in-depth research-gap paragraph.

Response: We sincerely thank Reviewer #3 for encouraging a more explicit articulation of the research gap. Section 2.3 already identifies four distinct limitations in the existing literature. We added a concise synthesis paragraph that states the central unresolved issue more directly: not merely whether retail investors adopt or trust AI-supported advisory services, but how such trust is translated into capability-relevant behavior and under what protective conditions this conversion is more likely to occur.

Comment 6 – Compare the findings with researchers from other countries.

Response: We sincerely thank Reviewer #3 for suggesting a comparison with evidence from other countries. We added a focused comparison in the Discussion with Malaysian evidence [3] and Indian evidence [20], [21]. The new paragraph explains both the consistency of the Vietnamese findings with

prior emerging-market studies and the additional contribution of testing the trust-to-behavior-to-capability pathway in the Vietnamese context.

Comment 7 – Segregate conclusion, policy implications, and future studies.

Response: We sincerely thank Reviewer #3 for recommending a clearer separation of the conclusion, policy implications, and future research. The manuscript already contained separate Conclusion and Future Research sections. We added a distinct subsection, "6.6 Policy Implications," retained Section 7 as the Conclusion, and revised Section 8 to "Limitations and Future Research Directions." This structure now separates the three elements clearly while avoiding unnecessary restructuring of the manuscript.

We again sincerely thank the Academic Editor and all Reviewers for their careful and constructive comments. We believe that these focused revisions have improved the clarity, methodological transparency, theoretical positioning, and reproducibility of the manuscript while preserving the original study design, model, sample, and reported results.
