# Supplementary material for: Digital financial literacy and trust in hybrid AI advisory: Explaining perceived private wealth management capability among digitally engaged retail investors in an emerging market
Source: PLoS One. 2026 Oct 30;21(10):e0353922. doi: 10.1371/journal.pone.0353922 (PMC13722208; doi:10.1371/journal.pone.0353922)
Supplement: Supplementary file 3 [file pone.0353922.s007.pdf]

Response to Academic Editor and Reviewers

Dear Academic Editor and Reviewers,

We sincerely thank the Academic Editor and Reviewers 3 and 4 for their careful evaluation of the revised manuscript. We appreciate the reviewers' assessment that the manuscript is technically sound, that the statistical analysis is appropriate, that the underlying data support the conclusions, and that it is written in clear English. In response to the additional minor comments from Reviewer #4, we have further revised the manuscript to strengthen the balance of recent literature, clarify the operationalization of hybrid AI advisory and respondent eligibility, improve measurement transparency, address reliability and indicator-overlap concerns, strengthen the interpretation of discriminant validity, make the practical and policy implications more actionable, maintain appropriate limits on generalization, and consolidate the study limitations. The conceptual model, hypotheses, sample (N = 249), measurement model, and reported structural results remained unchanged.

A. Academic Editor and Journal Requirements

Requirement 1 – Revision package and file preparation

We sincerely appreciate the Academic Editor's invitation to submit a revised version. In accordance with the journal's instructions, the revision package includes a point-by-point Response to Reviewers, a marked-up revised manuscript showing the changes, and a clean manuscript. The revision remains focused on the issues raised in the second-round decision letter.

Requirement 2 – Reference-list review

We reviewed the reference list again for completeness, consistency, relevance, and currency. Foundational theoretical sources were retained where necessary, while recent empirical evidence from 2023–2026 was given greater visibility in the literature review and discussion. No citation was added solely for compliance.

B. Reviewer #3

Comment – All comments have been addressed

Response: We sincerely thank Reviewer #3 for confirming that all previous comments have been addressed and for indicating that the manuscript is technically sound, the statistical analysis is appropriate, the underlying data are available, and the manuscript is clearly written. As Reviewer #3 provided no additional comments in this round, no further reviewer-specific revision was required.

C. Reviewer #4

Comment 1 – Improve the literature review's balance between foundational and recent studies.

Response: We sincerely thank Reviewer #4 for this helpful suggestion. We further strengthened the balance between foundational theory and recent empirical evidence by giving greater visibility to

studies published during 2023–2026, including recent work on digital financial literacy, AI financial advisors, advisory transparency, and trust in robo-advisory. The revised Introduction and Literature Review now use recent evidence not only to demonstrate currency but also to identify where findings converge, diverge, and reveal the unresolved trust-to-behavior gap. The Discussion additionally compares the Vietnamese findings with evidence from Malaysia and India and explains where the findings are consistent with prior studies and where the present study extends them. Foundational sources are retained only where they are necessary to establish the original theoretical constructs.

Comment 2 – Clarify the operationalization of "hybrid AI advisory."

Response: We sincerely thank Reviewer #4 for emphasizing the need for a precise operational definition. The revised Introduction now defines hybrid AI advisory as a wealth-management service configuration in which artificial intelligence generates or supports investment recommendations. At the same time, human advisors provide complementary interpretation, oversight, or personalized guidance. This definition explicitly distinguishes the study context from fully automated robo-advisory. We also clarified the eligibility criteria in Section 3.1 and the screening procedure in Section 3.2. Eligible respondents were required to have prior exposure to a digital financial platform, investment application, robo-advisory tool, or AI-supported advisory function, as well as experience in monitoring, allocating, or adjusting personal financial assets. These criteria were used to ensure that respondents could meaningfully evaluate the hybrid AI advisory context.

Comment 3 – Provide greater detail on questionnaire adaptation.

Response: We sincerely thank Reviewer #4 for this constructive recommendation. To improve transparency and reproducibility, we added Table 1, "Measurement Sources and Adaptation Procedures," which identifies each construct, its measurement source, the number of items, the adaptation procedure, and an example item. Section 3.2 also explains the adaptation process in greater detail: construct-specific items were adapted from published sources, their original conceptual meanings were retained, wording was contextualized to hybrid AI-supported wealth management, the English and Vietnamese versions were compared item by item for semantic consistency, and the instrument was pilot-tested with 10 eligible Vietnamese individual investors. No item was removed after the pilot test.

Comment 4 – Reconsider the reporting of extremely high reliability coefficients.

Response: We sincerely thank Reviewer #4 for drawing attention to the upper range of the reliability coefficients. We revised the measurement-model discussion to explicitly acknowledge this issue. Composite reliability values range from 0.898 to 0.948; all values exceed the recommended minimum and remain below the 0.95 upper benchmark. We therefore interpret the results as satisfactory internal consistency while noting that values approaching 0.95 warrant attention to possible item redundancy.

The construct items were retained because they represent the intended content domains, and the combined assessment of conceptual coverage, indicator wording, and collinearity diagnostics does not suggest substantial redundancy.

Comment 5 – Explain the relatively high outer loadings.

Response: We sincerely thank Reviewer #4 for this important observation. We reviewed the wording and conceptual content of the indicators in light of the high outer loadings (0.846–0.940). The revised discussion of the measurement clarifies that the strong loadings indicate high indicator reliability. At the same time, the measurement source and adaptation table demonstrate that the indicators were contextualized to capture distinct facets within their respective constructs rather than repeated wording. We also retained the indicator-level collinearity assessment; the outer VIF values remain below the critical threshold of 5.00. On this basis, the high loadings were not treated as grounds for indicator deletion, as removing indicators solely to reduce loadings could risk weakening construct content validity.

Comment 6 – Improve the interpretation of discriminant validity.

Response: We sincerely thank Reviewer #4 for requesting a clearer interpretation of the relationship between digital financial literacy (DFL) and trust in hybrid AI advisory (THA). The revised manuscript distinguishes the constructs conceptually: DFL represents a user-side capability to access, understand, evaluate, and use digital financial information, whereas THA represents confidence in the hybrid advisory system's reliability and competence. They are therefore theoretically related but conceptually different: DFL reflects users' ability to evaluate digital financial information, whereas THA reflects their willingness to rely on the advisory system. Empirically, the highest HTMT value is 0.743 for DFL–THA, below the conservative 0.85 threshold; the corresponding bootstrapped upper confidence bound is 0.823, also below 0.85. The Fornell–Larcker results provide additional evidence of empirical distinctness. We have clarified this interpretation while retaining the substantive relationship between literacy and informed trust.

Comment 7 – Improve the practical implications.

Response: We sincerely thank Reviewer #4 for encouraging more actionable implications. Section 6 has been strengthened substantially. The revised manuscript recommends capability-based digital financial literacy modules, understandable and decision-relevant AI explanations, scenario analysis and risk–return visualizations, portfolio-monitoring alerts and progress dashboards, contextual cyber-risk warnings and phishing-recognition exercises, multi-factor authentication prompts, opportunities for human consultation for higher-stakes decisions, and segmented support based on users' literacy, behavioral engagement, and cyber-risk self-protection. Section 6.6 also recommends proportionate disclosure standards for AI-supported financial advice, digital financial literacy, and cybersecurity

education, as well as continued access to human review. These recommendations are framed as enabling safeguards rather than claims about objective investment performance.

Comment 8 – Avoid overgeneralization.

Response: We sincerely thank Reviewer #4 for this important reminder. We reviewed the Abstract, methodology, Discussion, Conclusion, policy implications, and limitations to ensure that the population boundary is stated consistently. The manuscript now repeatedly specifies that the evidence pertains to digitally engaged retail investors in Vietnam and should not be generalized to all Vietnamese investors, private banking clients, high-net-worth individuals, objective portfolio performance, or verified wealth outcomes. Perceived Private Wealth Management Capability is consistently described as a self-assessed capability outcome rather than an objective performance measure.

Comment 9 – Strengthen the limitations section.

Response: We sincerely thank Reviewer #4 for recommending a more systematic discussion of limitations. Section 8, "Limitations and Future Research Directions," now explicitly integrates all of the requested limitations: non-probability convenience sampling, the relatively small sample size, the cross-sectional design, reliance on self-reported measures, the possibility of common method variance, the Vietnam-specific context, the self-assessed nature of perceived private wealth management capability rather than objective financial outcomes, and potential selection bias arising from the digitally engaged respondent profile. The revised section also recommends longitudinal designs, objective investment indicators, cross-country samples, and experimental approaches for future research.

We again sincerely thank the Academic Editor and Reviewers 3 and 4 for their careful and constructive evaluation. We believe that these focused second-round revisions have improved the manuscript's conceptual precision, measurement transparency, interpretation, practical relevance, and reporting of limitations while preserving the original study design, sample, model, and analytical results.
