# Supplementary material for: Digital financial literacy and trust in hybrid AI advisory: Explaining perceived private wealth management capability among digitally engaged retail investors in an emerging market
Source: PLoS One. 2026 Oct 30;21(10):e0353922. doi: 10.1371/journal.pone.0353922 (PMC13722208; doi:10.1371/journal.pone.0353922)
Supplement: Peer review history

# Peer review: Decision Letter 0

**DOI:** 10.1371/journal.pone.0353922.r001

**Authors:** Sajid Mohy Ul Din

13 Aug 2026

DIGITAL FINANCIAL LITERACY AND TRUST IN HYBRID AI ADVISORY: EXPLAINING PERCEIVED PRIVATE WEALTH MANAGEMENT CAPABILITY AMONG DIGITALLY ENGAGED RETAIL INVESTORS IN AN EMERGING MARKET

PLOS One

Dear Dr. Tran,

Thank you for submitting your manuscript to PLOS ONE. After careful consideration, we feel that it has merit but does not fully meet PLOS ONE’s publication criteria as it currently stands. Therefore, we invite you to submit a revised version of the manuscript that addresses the points raised during the review process.

Please submit your revised manuscript by Sep 11 2026 11:59PM. If you will need more time than this to complete your revisions, please reply to this message or contact the journal office at plosone@plos.org. When you're ready to submit your revision, log on to https://www.editorialmanager.com/pone/ and select the 'Submissions Needing Revision' folder to locate your manuscript file.

Please include the following items when submitting your revised manuscript:

- A letter that responds to each point raised by the academic editor and reviewer(s). You should upload this letter as a separate file labeled 'Response to Reviewers'.
- A marked-up copy of your manuscript that highlights changes made to the original version. You should upload this as a separate file labeled 'Revised Manuscript with Track Changes'.
- An unmarked version of your revised paper without tracked changes. You should upload this as a separate file labeled 'Manuscript'.

If you would like to make changes to your financial disclosure, please include your updated statement in your cover letter. Guidelines for resubmitting your figure files are available below the reviewer comments at the end of this letter.

If applicable, we recommend that you deposit your laboratory protocols in protocols.io to enhance the reproducibility of your results. Protocols.io assigns your protocol its own identifier (DOI) so that it can be cited independently in the future. For instructions see: https://journals.plos.org/plosone/s/submission-guidelines#loc-laboratory-protocols. Additionally, PLOS ONE offers an option for publishing peer-reviewed Lab Protocol articles, which describe protocols hosted on protocols.io. Read more information on sharing protocols at https://plos.org/protocols?utm_medium=editorial-email&utm_source=authorletters&utm_campaign=protocols.

As the corresponding author, your ORCID iD is verified in the submission system and will appear in the published article. PLOS supports the use of ORCID, and we encourage all coauthors to register for an ORCID iD and use it as well. Please encourage your coauthors to verify their ORCID iD within the submission system before final acceptance, as unverified ORCID iDs will not appear in the published article. *Only*  the individual author can complete the verification step; PLOS staff *cannot*  verify ORCID iDs on behalf of authors.

We look forward to receiving your revised manuscript.

Kind regards,

Sajid Mohy Ul Din

Academic Editor

PLOS One

Journal Requirements:

When submitting your revision, we need you to address these additional requirements.

1.Please ensure that your manuscript meets PLOS ONE's style requirements, including those for file naming. The PLOS ONE style templates can be found at

https://journals.plos.org/plosone/s/file?id=wjVg/PLOSOne_formatting_sample_main_body.pdf and

https://journals.plos.org/plosone/s/file?id=ba62/PLOSOne_formatting_sample_title_authors_affiliations.pdf

2. Thank you for stating the following financial disclosure:

“This research is partly funded by Ho Chi Minh City University of Economics and Finance, Ho Chi Minh City, Vietnam.”

Please state what role the funders took in the study.  If the funders had no role, please state: 'The funders had no role in study design, data collection and analysis, decision to publish, or preparation of the manuscript.'

If this statement is not correct you must amend it as needed.

Please include this amended Role of Funder statement in your cover letter; we will change the online submission form on your behalf.

3. We note you have included a table to which you do not refer in the text of your manuscript. Please ensure that you refer to Tables 1, 2, 3, 4, 5, 6, 8, 9, 10, 11, 12, 13, and 14 in your text; if accepted, production will need this reference to link the reader to the Table.

4. Please include captions for your Supporting Information files at the end of your manuscript, and update any in-text citations to match accordingly. Please see our Supporting Information guidelines for more information: http://journals.plos.org/plosone/s/supporting-information.

5. If the reviewer comments include a recommendation to cite specific previously published works, please review and evaluate these publications to determine whether they are relevant and should be cited. There is no requirement to cite these works unless the editor has indicated otherwise.

6. Please review your reference list to ensure that it is complete and correct. If you have cited papers that have been retracted, please include the rationale for doing so in the manuscript text, or remove these references and replace them with relevant current references. Any changes to the reference list should be mentioned in the rebuttal letter that accompanies your revised manuscript. If you need to cite a retracted article, indicate the article’s retracted status in the References list and also include a citation and full reference for the retraction notice.

Additional Editor Comments:

Dear author (s), please share the study's questionnaire either as supplementary material or in article

[Note: HTML markup is below. Please do not edit.]

Reviewer's Responses to Questions

**Comments to the Author**

1. Is the manuscript technically sound, and do the data support the conclusions?

Reviewer #1: Yes

Reviewer #2: Yes

Reviewer #3: Yes

**********

2. Has the statistical analysis been performed appropriately and rigorously?

Reviewer #1: Yes

Reviewer #2: Yes

Reviewer #3: Yes

**********

3. Have the authors made all data underlying the findings in their manuscript fully available?

The PLOS Data policy

Reviewer #1: No

Reviewer #2: Yes

Reviewer #3: Yes

**********

4. Is the manuscript presented in an intelligible fashion and written in standard English?

Reviewer #1: Yes

Reviewer #2: Yes

Reviewer #3: Yes

**********

Reviewer #1: Make sure to run Harman’s single-factor test or report full collinearity VIFs (ensuring values are under 3.3 in SmartPLS)

Reviewer #2: The study is well prepared and demonstrates several strengths. It combines scientific rigor in addressing the research topic with its practical application through the field study and the selected research sample.

Nevertheless, the following comments should be taken into consideration:

1.The methodological procedures of the field study should be described in greater detail.

2.The source on which the questionnaire was based should be clearly identified, and the rationale for adopting it should be provided.

3. A section on the internal consistency validity of the research instrument (the questionnaire) should be included (Page 29). This section should explain that the questionnaire was submitted to a panel of experts for review and validation, and that, in light of their comments and recommendations, the instrument was revised and finalized before being administered to the study sample.

4.The discussion of the hypothesis testing results should be expanded and supported by a comparison with the findings of the previous studies reviewed in the literature.

Reviewer #3: I read your work about DIGITAL FINANCIAL LITERACY AND TRUST IN HYBRID AI ADVISORY: EXPLAINING PERCEIVED PRIVATE WEALTH MANAGEMENT CAPABILITY AMONG DIGITALLY ENGAGED RETAIL INVESTORS IN AN EMERGING MARKET, and I sincerely congratulate the author(s) for conducting research on such an important topic. However, the author(s) may improve some areas for better clarity. Here are some minor comments on this matter:

The objective of the study is not clear.

Please provide a clear, in-depth objective.

The authors need to provide some theoretical support in the Introduction and Literature Review section.

The authors need to provide the latest citations [2020-2025] in their literature review section.

The authors need to provide an in-depth paragraph related to the research gap in the Literature Review section

The authors need to provide some theoretical support in the Introduction and Literature Review.

The authors need to compare their results with other countries ' researchers' results on a similar topic.

The authors should segregate the conclusion, policy implications, and scope for further studies.

Best wishes,

**********

what does this mean?). If published, this will include your full peer review and any attached files.

If you choose “no”, your identity will remain anonymous but your review may still be made public.

**Do you want your identity to be public for this peer review?**  For information about this choice, including consent withdrawal, please see our Privacy Policy

Reviewer #1: **Yes:**  Assist. prof. Yaamr Abdulateef Hussein

Reviewer #2: **Yes:**  ..

Reviewer #3: No

**********

[NOTE: If reviewer comments were submitted as an attachment file, they will be attached to this email and accessible via the submission site. Please log into your account, locate the manuscript record, and check for the action link "View Attachments". If this link does not appear, there are no attachment files.]

To ensure your figures meet our technical requirements, please review our figure guidelines: https://journals.plos.org/plosone/s/figures

You may also use PLOS’s free figure tool, NAAS, to help you prepare publication quality figures: https://journals.plos.org/plosone/s/figures#loc-tools-for-figure-preparation.

NAAS will assess whether your figures meet our technical requirements by comparing each figure against our figure specifications.

# Author comment: Author response to Decision Letter 1

**DOI:** 10.1371/journal.pone.0353922.r002

17 Aug 2026

Please see the attached point-by-point Response to Reviewers file for our detailed responses to the Academic Editor and all reviewers.

Attachment Submitted filename: Response to Reviewers.pdf

# Peer review: Decision Letter 1

**DOI:** 10.1371/journal.pone.0353922.r003

**Authors:** Sajid Mohy Ul Din

14 Sep 2026

Digital financial literacy and trust in hybrid AI advisory: Explaining perceived private wealth management capability among digitally engaged retail investors in an emerging market

PLOS One

Dear Dr. Tran,

Thank you for submitting your manuscript to PLOS One. After careful consideration, we feel that it has merit but does not fully meet PLOS One’s publication criteria as it currently stands. Therefore, we invite you to submit a revised version of the manuscript that addresses the points raised during the review process.

Please submit your revised manuscript by Oct 29 2026 11:59PM. If you will need more time than this to complete your revisions, please reply to this message or contact the journal office at plosone@plos.org. When you're ready to submit your revision, log on to https://www.editorialmanager.com/pone/ and select the 'Submissions Needing Revision' folder to locate your manuscript file.

- A letter that responds to each point raised by the academic editor and reviewer(s). You should upload this letter as a separate file labeled 'Response to Reviewers'.
- A marked-up copy of your manuscript that highlights changes made to the original version. You should upload this as a separate file labeled 'Revised Manuscript with Track Changes'.
- An unmarked version of your revised paper without tracked changes. You should upload this as a separate file labeled 'Manuscript'.

If you would like to make changes to your financial disclosure, please include your updated statement in your cover letter. Guidelines for resubmitting your figure files are available below the reviewer comments at the end of this letter.

If applicable, we recommend that you deposit your laboratory protocols in protocols.io to enhance the reproducibility of your results. Protocols.io assigns your protocol its own identifier (DOI) so that it can be cited independently in the future. For instructions see: https://journals.plos.org/plosone/s/submission-guidelines#loc-laboratory-protocols. Additionally, PLOS One offers an option for publishing peer-reviewed Lab Protocol articles, which describe protocols hosted on protocols.io. Read more information on sharing protocols at https://plos.org/protocols?utm_medium=editorial-email&utm_source=authorletters&utm_campaign=protocols.

As the corresponding author, your ORCID iD is verified in the submission system and will appear in the published article. PLOS supports the use of ORCID, and we encourage all coauthors to register for an ORCID iD and use it as well. Please encourage your coauthors to verify their ORCID iD within the submission system before final acceptance, as unverified ORCID iDs will not appear in the published article. *Only*  the individual author can complete the verification step; PLOS staff *cannot*  verify ORCID iDs on behalf of authors.

We look forward to receiving your revised manuscript.

Kind regards,

Sajid Mohy Ul Din

Academic Editor

PLOS One

Journal Requirements:

If the reviewer comments include a recommendation to cite specific previously published works, please review and evaluate these publications to determine whether they are relevant and should be cited. There is no requirement to cite these works unless the editor has indicated otherwise.

Please review your reference list to ensure that it is complete and correct. If you have cited papers that have been retracted, please include the rationale for doing so in the manuscript text, or remove these references and replace them with relevant current references. Any changes to the reference list should be mentioned in the rebuttal letter that accompanies your revised manuscript. If you need to cite a retracted article, indicate the article’s retracted status in the References list and also include a citation and full reference for the retraction notice.

[Note: HTML markup is below. Please do not edit.]

Reviewers' comments:

Reviewer's Responses to Questions

**Comments to the Author**

Reviewer #3: All comments have been addressed

Reviewer #4: All comments have been addressed

**********

2. Is the manuscript technically sound, and do the data support the conclusions?

Reviewer #3: Yes

Reviewer #4: Yes

**********

3. Has the statistical analysis been performed appropriately and rigorously?

Reviewer #3: Yes

Reviewer #4: Yes

**********

4. Have the authors made all data underlying the findings in their manuscript fully available?

The PLOS Data policy

Reviewer #3: Yes

Reviewer #4: Yes

**********

5. Is the manuscript presented in an intelligible fashion and written in standard English?

Reviewer #3: Yes

Reviewer #4: Yes

**********

Reviewer #3: (No Response)

Reviewer #4: Minor Comments

1. Improve the literature review's balance between foundational and recent studies.

The manuscript includes both foundational theoretical sources and recent empirical studies, which is appropriate. Nevertheless, because AI-supported financial advisory is a rapidly developing field, the literature review should continue to prioritize recent empirical evidence, particularly studies published during 2023–2026.

Recent literature should be used not merely to demonstrate currency but to identify where findings converge or conflict.

2. Clarify the operationalization of “hybrid AI advisory.”

The term “hybrid AI advisory” is central to the manuscript, but readers would benefit from a more precise operational definition.

Please clarify whether hybrid AI advisory refers to:

AI plus human financial advisors;

AI-generated recommendations reviewed by humans;

robo-advisory platforms with optional human intervention; or

another specific configuration.

The eligibility criteria should also explain how respondents were determined to have sufficient exposure to hybrid AI advisory.

3. Provide greater detail on questionnaire adaptation.

The authors explain that items were adapted from multiple published sources and contextualized for hybrid AI-supported wealth management.

This is appropriate, but a table identifying each construct, original source, number of items, adaptation process, and example item would substantially improve transparency and reproducibility.

4. Reconsider the reporting of extremely high reliability coefficients.

The reported composite reliability values range from 0.898 to 0.948. Although these values are technically acceptable, values approaching 0.95 can sometimes indicate item redundancy.

The authors should briefly discuss whether the high reliability coefficients indicate that some items may be conceptually overlapping.

5. Explain the relatively high outer loadings.

The outer loadings range from 0.846 to 0.940.

While these values demonstrate strong indicator reliability, the authors should ensure that the indicators are not excessively similar in wording or conceptual content.

6. Improve the interpretation of discriminant validity.

The HTMT values are below 0.85, with the highest value being 0.743 between digital financial literacy and trust in hybrid AI advisory.

The results are satisfactory, but because these two constructs are theoretically related, the authors should provide a brief explanation of how they differ conceptually and empirically.

7. Improve the practical implications.

The policy and managerial implications are relevant, particularly for financial institutions, fintech providers, and investor education programs.

However, the implications could be made more actionable by identifying specific interventions, such as:

digital financial literacy modules;

explainable AI interfaces;

investor risk alerts;

cyber-risk awareness mechanisms;

human-in-the-loop advisory systems; and

regulatory guidance for AI-supported investment advice.

8. Avoid overgeneralization.

The manuscript appropriately acknowledges that the sample represents digitally engaged retail investors rather than all Vietnamese investors, private banking clients, or high-net-worth individuals.

This limitation should also be consistently reflected in the abstract, discussion, conclusion, and policy implications.

9. Strengthen the limitations section.

The limitations section should explicitly include:

non-probability sampling;

relatively small sample size;

cross-sectional design;

self-reported measures;

potential common method variance;

country-specific context;

self-assessed capability rather than objective financial outcomes; and

potential selection bias arising from digitally engaged respondents.

The manuscript already addresses several of these limitations, but they could be integrated into a more systematic discussion.

**********

what does this mean?). If published, this will include your full peer review and any attached files.

If you choose “no”, your identity will remain anonymous but your review may still be made public.

**Do you want your identity to be public for this peer review?**  For information about this choice, including consent withdrawal, please see our Privacy Policy

Reviewer #3: No

Reviewer #4: No

**********

[NOTE: If reviewer comments were submitted as an attachment file, they will be attached to this email and accessible via the submission site. Please log into your account, locate the manuscript record, and check for the action link "View Attachments". If this link does not appear, there are no attachment files.]

To ensure your figures meet our technical requirements, please review our figure guidelines: https://journals.plos.org/plosone/s/figures

You may also use PLOS’s free figure tool, NAAS, to help you prepare publication quality figures: https://journals.plos.org/plosone/s/figures#loc-tools-for-figure-preparation.

NAAS will assess whether your figures meet our technical requirements by comparing each figure against our figure specifications.

# Author comment: Author response to Decision Letter 2

**DOI:** 10.1371/journal.pone.0353922.r004

21 Sep 2026

We sincerely thank the Academic Editor and the reviewers for their valuable comments and constructive suggestions. We have carefully revised the manuscript and addressed all comments point by point. A detailed response to each comment has been provided in the uploaded Response to Reviewers file. All revisions are highlighted in the marked-up manuscript, and a clean revised manuscript has also been submitted.

We appreciate the opportunity to revise our manuscript and hope that the revised version meets the journal's requirements.

Attachment Submitted filename: Response_to_Reviewers_auresp_2.pdf

# Peer review: Decision Letter 2

**DOI:** 10.1371/journal.pone.0353922.r005

**Authors:** Sajid Mohy Ul Din

20 Oct 2026

Digital financial literacy and trust in hybrid AI advisory: Explaining perceived private wealth management capability among digitally engaged retail investors in an emerging market

PONE-D-26-32079R2

Dear Dr. Tran,

We’re pleased to inform you that your manuscript has been judged scientifically suitable for publication and will be formally accepted for publication once it meets all outstanding technical requirements.

Within one week, you’ll receive an e-mail detailing the required amendments. When these have been addressed, you’ll receive a formal acceptance letter and your manuscript will be scheduled for publication.

An invoice will be generated when your article is formally accepted. Please note, if your institution has a publishing partnership with PLOS and your article meets the relevant criteria, all or part of your publication costs will be covered. Please make sure your user information is up-to-date by logging into Editorial Manager at Editorial Manager® and clicking the ‘Update My Information' link at the top of the page. For questions related to billing, please contact billing support.

If your institution or institutions have a press office, please notify them about your upcoming paper to help maximize its impact. If they’ll be preparing press materials, please inform our press team as soon as possible -- no later than 48 hours after receiving the formal acceptance. Your manuscript will remain under strict press embargo until 2 pm Eastern Time on the date of publication. For more information, please contact onepress@plos.org.

Kind regards,

Sajid Mohy Ul Din

Academic Editor

PLOS One

**********

# Editor report: Acceptance letter

**DOI:** 10.1371/journal.pone.0353922.r006

**Authors:** Sajid Mohy Ul Din

PONE-D-26-32079R2

PLOS One

Dear Dr. Tran,

I'm pleased to inform you that your manuscript has been deemed suitable for publication in PLOS One. Congratulations! Your manuscript is now being handed over to our production team.

At this stage, our production department will prepare your paper for publication. This includes ensuring the following:

* All references, tables, and figures are properly cited

* All relevant supporting information is included in the manuscript submission,

* There are no issues that prevent the paper from being properly typeset

You will receive further instructions from the production team, including instructions on how to review your proof when it is ready. Please keep in mind that we are working through a large volume of accepted articles, so please give us a few days to review your paper and let you know the next and final steps.

Lastly, if your institution or institutions have a press office, please let them know about your upcoming paper now to help maximize its impact. If they'll be preparing press materials, please inform our press team within the next 48 hours. Your manuscript will remain under strict press embargo until 2 pm Eastern Time on the date of publication. For more information, please contact onepress@plos.org.

You will receive an invoice from PLOS for your publication fee after your manuscript has reached the completed accept phase. If you receive an email requesting payment before acceptance or for any other service, this may be a phishing scheme. Learn how to identify phishing emails and protect your accounts at https://explore.plos.org/phishing.

If we can help with anything else, please email us at customercare@plos.org.

Thank you for submitting your work to PLOS One and supporting open access.

Kind regards,

PLOS One Editorial Office Staff

on behalf of

Dr. Sajid Mohy Ul Din

Academic Editor

PLOS One
